# Label-free target identification using in-gel fluorescence difference *via* thermal stability shift†

**DOI:** 10.1039/c6sc03238a

**Published:** 2016-09-22

**Authors:** Hankum Park, Jaeyoung Ha, Ja Young Koo, Jongmin Park, Seung Bum Park

**Affiliations:** a Department of Biophysics and Chemical Biology, Seoul National University Seoul – 08826 Korea sbpark@snu.ac.kr +82-2-884-4025; b CRI Center for Chemical Proteomics, Department of Chemistry, Seoul National University Seoul – 08826 Korea

## Abstract

Target engagement is a prerequisite for the therapeutic effects of bioactive small molecules, and unbiased identification of their target proteins can facilitate drug discovery and chemical biology research. Structural modifications of bioactive natural products for target identification exhibit potential limitations such as synthetic difficulties, limited supplies from natural sources, and loss of original efficacy. Herein, we developed a label-free method for proteome-wide target identification using in-gel fluorescence difference caused by thermal stability shift, namely TS-FITGE. Quantitative intra-gel image analysis of each protein spot revealed target proteins with shifted thermal stability upon drug engagement, and plotting of melting curves by inter-gel analysis confirmed the positive targets. We demonstrated the robustness and applicability of the TS-FITGE method by identifying target proteins, including membrane-anchored proteins, of complex bioactive compounds. Furthermore, we identified and functionally validated nucleophosmin as a novel target protein of hordenine, a natural product upregulator of *in vitro* translation.

## Abstract 2

A label-free method for proteome-wide target identification was developed using in-gel fluorescence difference caused by thermal stability shift.

## Graphical abstract

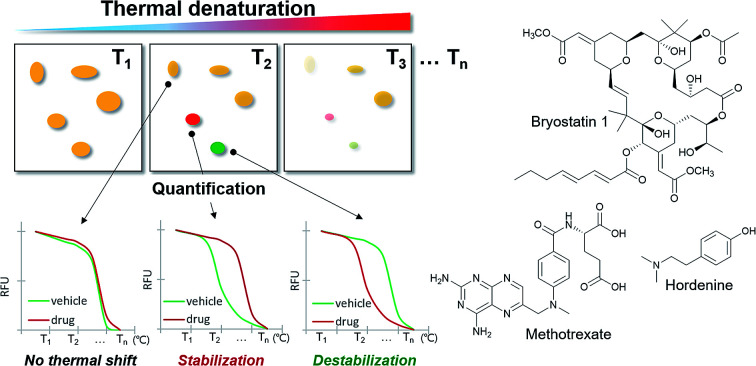

## Introduction

In order for small molecules to exert their biological activities, they should bind to their biomolecular partners. This event is known as target engagement, an important aspect of drug discovery and chemical biology research.^1^ The target proteins of traditional natural products or hit compounds from phenotypic screening are unknown in most cases.^2,3^ Therefore, target proteins should be identified to determine the mechanisms of action of bioactive small molecules for the development of novel therapeutic agents or research tools in biomedical sciences.

The commonly used approach for target identification (ID) includes the affinity chromatography-pull down assay,^4^ activity-based proteome profiling,^5^ and affinity-based proteome profiling.^2^ In most cases, chemical modification of original bioactive compounds was required to introduce functional handles such as electrophiles for the enzymatic reaction, alkyne moieties for click chemistry, or photoaffinity groups for covalent crosslinking to target proteins. The design and synthesis for the functionalization, however, remain as major hurdles in the target ID because a great deal of effort is inevitable to synthesize numerous analogues and explore their structure–activity relationships.^6^ In fact, there were many unsuccessful attempts in preparing target ID probes with retained bioactivities because even slight modifications may abolish the biological activity of original hit compounds. Cumbersome total syntheses of complex natural products and their insufficient extraction from natural sources also limit the access to target ID probes. Therefore, a label-free target ID method without any structural modification is in high demand.

In the recently reported cellular thermal stability assay (CETSA), target engagement was monitored in a label-free manner in intact cells or tissues.^7^ The amount of proteins of interest in the soluble fraction was measured by western blot analysis after thermal denaturation. The engagement of bioactive ligands stabilized target proteins against heat denaturation and increased the melting temperature (*T*_m_) of the protein. Given its robustness and effectiveness, CETSA has been rapidly adopted by many researchers to confirm the target engagement.^8^ However, CETSA is not suitable for unbiased proteome-wide target ID because this method is only applicable to hypothesis-driven candidate proteins with available antibodies.

Unbiased target ID methods should be developed as a requisite for phenotypic screening, leading to the successful development of first-in-class therapeutics.^9^ In this study, we developed a label-free method for proteome-wide target ID using thermal stability shift-based fluorescence difference in two-dimensional gel electrophoresis (TS-FITGE) and demonstrated the robustness and practicality of the TS-FITGE method *via* target ID of bioactive natural products with extreme chemical structures: a complex natural product that is difficult to be synthesized and a simple natural product lacking room for chemical modification. TS-FITGE method successfully revealed the known target proteins of methotrexate and bryostatin 1, confirming the applicability of this method in the identification of membrane-anchored proteins. Furthermore, the unknown target protein of hordenine, a simple natural product that upregulates *in vitro* protein translation, was also identified and functionally validated.

## Results and discussion

### Strategy for a proteome-wide target ID using thermal stability shift

**Fig. 1:**
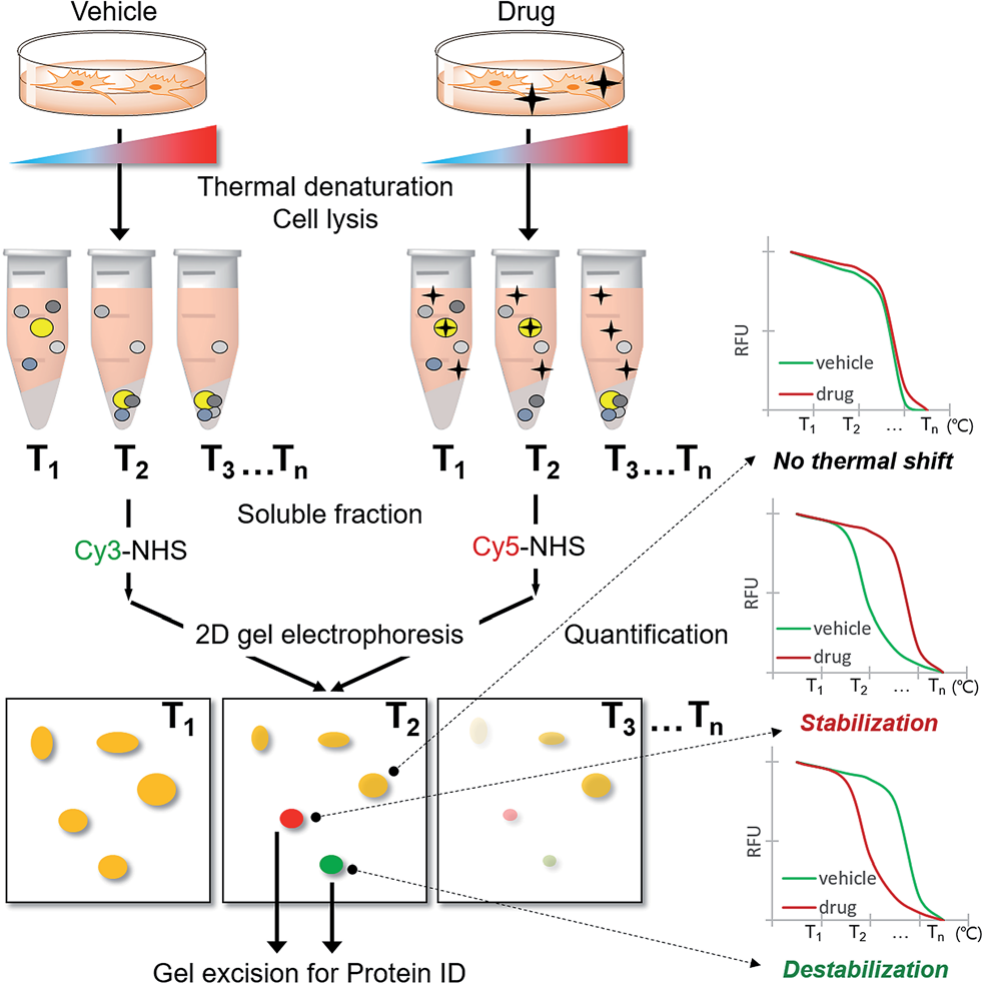
Scheme of the TS-FITGE experiment. Briefly, the target protein (colored as bright yellow in the microtube) was thermally stabilized by drug engagement and remained in the soluble fraction after thermal denaturation. The soluble proteome of the vehicle-treated group was conjugated to Cy3 dye and the drug-treated group to Cy5 dye, and the two samples were analyzed by 2D gel electrophoresis. The red and green colored spots indicated the thermal stability shift. The melting curves of each spot were plotted against various temperatures. Candidate protein spots were excised and identified by mass spectrometry.

As CETSA is applicable for biased candidate proteins using designated antibodies, we applied the thermal stability shift to our FITGE technique^10^ for proteome-wide target ID. As shown in Fig. 1, cells were heated at a range of temperatures to induce thermal denaturation. Most proteins would have the same denaturation patterns in the absence or presence of the drug, whereas target proteins with shifted thermal stability would show different amounts in the soluble fraction upon drug engagement. After centrifugation, all proteins in the soluble fraction were conjugated with two different dyes containing *N*-hydroxysuccinimide (NHS) ester: Cy3-NHS for the vehicle-treated group and Cy5-NHS for the drug-treated group. The dye-conjugated proteomes of both groups were mixed and separated by 2D gel electrophoresis (2-DE), and the ratio of Cy5 to Cy3 fluorescence signal of each protein spot was quantified by automated image analysis. Proteins that did not undergo thermal stability shift by the drug appeared as yellow spots (additive signals of Cy3 and Cy5), whereas proteins that were thermally stabilized and destabilized by drug engagement appeared as red spots and green spots, respectively. Additionally, the unheated proteome was conjugated with Cy2-NHS, and the same amount of Cy2-conjugated proteome was added to each gel sample as an internal standard for inter-gel quantification; therefore, the melting curves of each protein spot were obtained. Thermally shifted spots in TS-FITGE were considered to be potential target proteins and excised for identification by mass spectrometry.

### Preparation of three fluorescent dyes having the same electrophoretic mobility

To minimize deviations in mobility among Cy2-, Cy3-, and Cy5-conjugated proteins on 2D gels, we prepared charge- and mass-matched dyes (ESI Notes†); all dyes had +1 charge and differed by only 2 Da from each other (Fig. S1†). As the proteins conjugated with Cy2, Cy3, and Cy5 had the same mobility, most protein spots in the 37 °C gel appeared as white after merging (Fig. S2a†). At higher temperature, thermally unstable proteins were denatured and disappeared; therefore, these spots appeared as a blue color because of the unheated internal standard conjugated to Cy2 dye (Fig. S2b†).

### Proof-of-concept target identification study with methotrexate

**Fig. 2:**
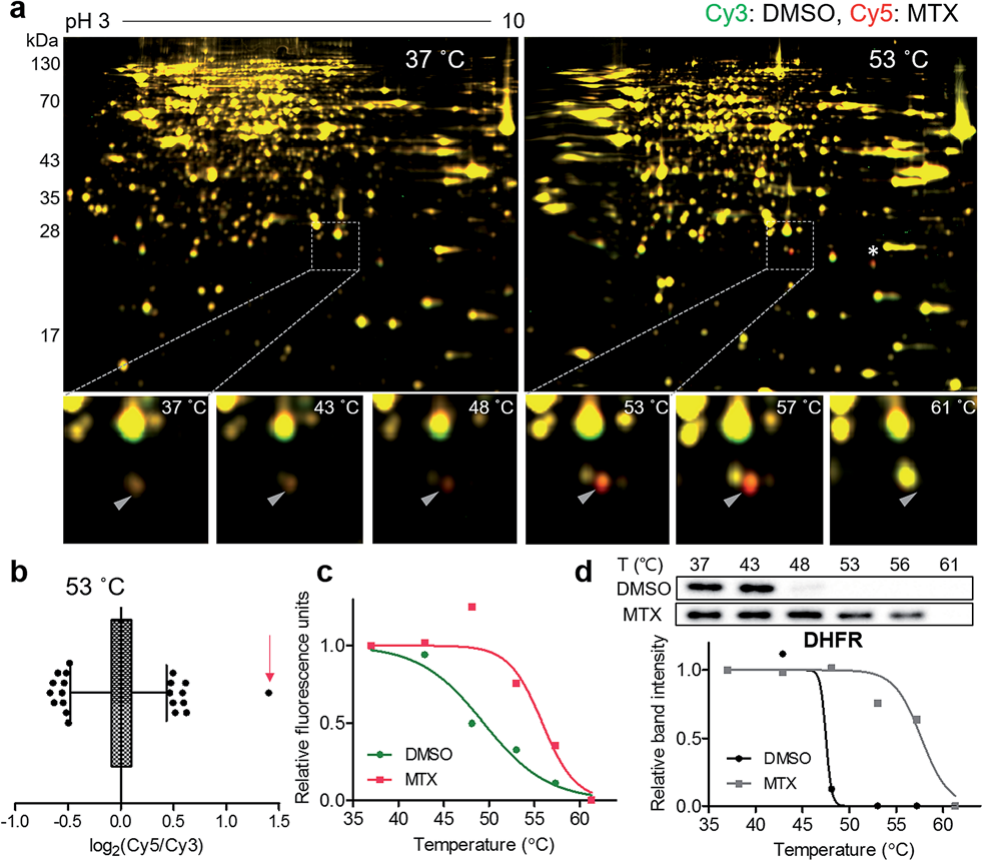
Unbiased identification of the target protein of methotrexate (MTX). (a) Representative images from TS-FITGE experiment. Images of Cy3 channel (green, proteome treated with DMSO) and Cy5 channel (red, proteome treated with MTX) were overlaid. (b) Box plot showing the distribution of Cy5/Cy3 fluorescence ratio for each spot in the 53 °C gel. The whiskers indicate 1–99 percentiles. The spot denoted by a triangle in (a) is indicated by a red arrow. (c) Melting curves showing a denaturation pattern of the indicated spot in (b). (d) Cellular thermal shift assay with dihydrofolate reductase (DHFR) antibody.

For a proof-of-concept investigation, methotrexate was selected because its major target protein, dihydrofolate reductase (DHFR), was known to have a significantly large *T*_m_ shift of 16 °C.^7^ Among a series of 2D gels at various denaturing temperatures, nearly all protein spots in the 37 °C gel were yellow, while a distinct red spot was detected in the 53 °C gel (Fig. 2a and S3†). For quantitative analysis, the ratios of Cy5 to Cy3 signals of all protein spots in the 53 °C gel were calculated and presented in a box plot (Fig. 2b). As expected, the red spot pointed by a triangle in Fig. 2a showed the highest ratio (indicated by an arrow in Fig. 2b), suggesting that this spot had significantly different Cy5 and Cy3 fluorescence signals. Although the spot indicated by an asterisk in Fig. 2a seemed as a colored spot, actually only the location of red and green spots were slightly discrepant from each other vertically, and the size and intensity of these spots were identical. The melting curve of the true red spot (pointed by a triangle in Fig. 2a) was plotted across a range of temperatures using Cy2 signals as internal standards and revealed a shift of the sigmoidal curve, indicating an increase in *T*_m_ for the potential target protein of methotrexate (Fig. 2c). Finally, the spot was excised and analyzed by LC-MS/MS, which revealed DHFR as the target protein (Table S1†). Western blot analysis using a monoclonal DHFR antibody revealed a comparable thermal shift pattern to the pattern determined by TS-FITGE (Fig. 2d). Moreover, thymidylate synthase, another target protein of methotrexate, was also identified as a thermal-stabilized spot showing a marked red color in the 48 °C gel, where the quantitative fluorescence difference of the thymidylate synthase spot was highest followed by the DHFR spot (Fig. S4 and Table S1†). As a result, we confirmed that TS-FITGE based on the thermal stability shift is a feasible and efficient strategy for unbiased target identification in live cells.

### Identification of membrane-anchored target proteins of bryostatin 1

**Fig. 3:**
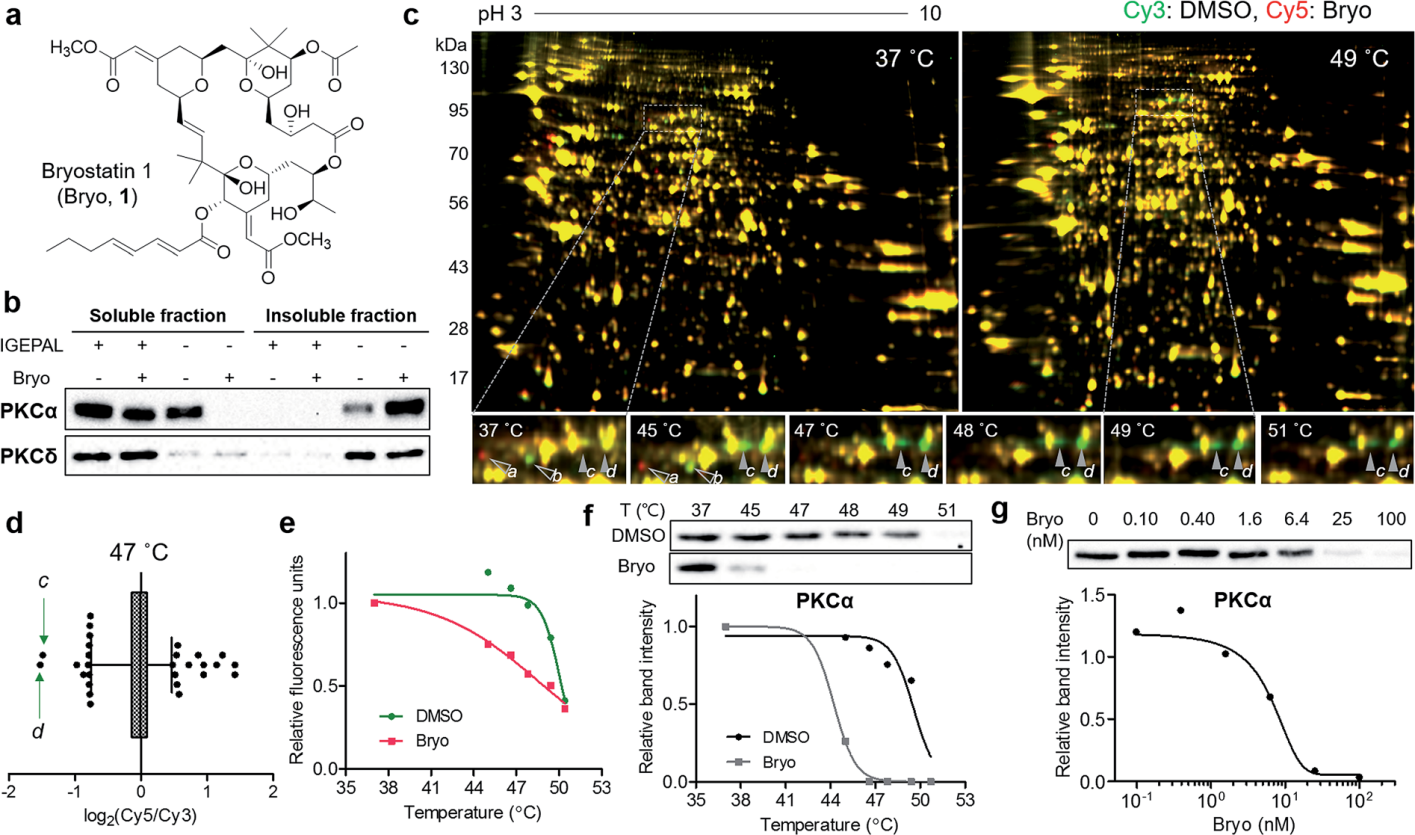
Thermal destabilization of the membrane-anchored target protein of bryostatin 1. (a) Chemical structure of bryostatin 1 (Bryo). (b) Translocation of PKC isozymes by Bryo and effect of IGEPAL CA-630 on solubilization of PKCs. (c) Representative images from TS-FITGE experiment. Images of Cy3 channel (green, proteome treated with DMSO) and Cy5 channel (red, proteome treated with Bryo) were overlaid. (d) Box plot showing the distribution of Cy5/Cy3 fluorescence ratio for each spot in the 47 °C gel. The whiskers indicate 1–99 percentiles. The protein spots labeled as *c* and *d* in (c) are indicated by green arrows. (e) Melting curves showing denaturation pattern of the protein spot *d*. (f) Immunoblot from cellular thermal shift assay with PKCα antibody. (g) An isothermal (48 °C) dose–response curve confirming thermal destabilization of PKCα by Bryo.

To confirm the usefulness of the TS-FITGE method, we identified the target protein of a complex natural product bryostatin 1. Bryostatins were first isolated from marine *Bugula neritina* and showed potent anticancer activity, synergistic chemotherapeutic activity, and memory-enhancing activity.^11^ After the structure of bryostatin 1 was elucidated in 1982,^12^ its 58-step total synthesis was reported in 2011.^13^ Therefore, bryostatin 1 is an example of a complex natural product for which it is difficult to synthesize appropriately functionalized probes for other target ID methods (Fig. 3a). Furthermore, bryostatins are known to modulate protein kinase Cs (PKCs) to translocate, anchor to the plasma membrane, and phosphorylate substrate proteins, which play important roles in cellular signaling processes.^14^ To investigate whether membrane-anchored proteins are compatible with our TS-FITGE method, we first screened the effect of detergents on solubilizing the PKCs by western blotting and optimized the lysis conditions in phosphate-buffered saline containing 0.4% (v/v) of IGEPAL CA-630, a nonionic and non-denaturing detergent, among various other detergents (Fig. S5a†). As shown in Fig. 3b, PKCα was mainly present in the soluble fraction before bryostatin treatment, but moved to insoluble fraction following treatment with bryostatin in lysis buffer without a mild detergent. In the presence of 0.4% of IGEPAL CA-630, however, PKCα remained in the soluble fraction even after bryostatin treatment. The location of PKCδ was not affected by bryostatin treatment, but a nonionic detergent was essential for solubilization in both cases (Fig. 3b). Solubilization of other PKC isozymes was not affected by bryostatin and detergent (Fig. S5b†). Thus, we concluded that 0.4% of IGEPAL CA-630 was required to globally solubilize PKCs and detect thermal stability shifts in the soluble fraction.

Next, we performed TS-FITGE experiment with bryostatin to monitor the thermal denaturation pattern of the cellular proteome using the optimized lysis conditions. Interestingly, several spots appeared as red or green not only in the heated samples but also in the unheated (37 °C) sample, indicating that the color difference in these spots did not originate from thermal shifting (Fig. 3c, protein *a* and *b*; Fig. S6,† protein *c*, *d*, *e*, *f*, *g*, *h*, *i*, and *j*). In addition, most red and green spots appeared horizontally next to each other in a pairwise arrangement. This phenomenon is a typical post-translational modification (PTM) pattern observed in 2-DE.^15^ Thus, we considered that these spots were not thermally shifted target proteins, but rather post-translationally modified downstream substrates after drug engagement at 37 °C. The red and green protein spots caused by PTM may be reduced simply by shortening the duration of drug treatment not to allow sufficient time for PTM to occur. As expected, the green spots *c* and *d* at 37 °C became yellowish, while the red spots including *e* disappeared as the treatment time decreased (Fig. S7a and b†). After identification of protein spots *c*, *d*, and *e* as PKCα (Table S1†), we performed western blot analysis using a PKCα antibody. The total amount of PKCα at 37 °C was identical in the 1D gel, but PKCα was separated to horizontal red and green spots in the 2D gel with increasing treatment time of bryostatin 1 (Fig. S7c and d†). These results were all consistent with the interpretation that the red and green spots in the 37 °C gel resulted from PTMs.

After 20 min treatment with bryostatin 1, we observed distinct green spots in the heated sample, but not in the unheated sample, indicating thermal destabilization upon target engagement (Fig. 3c, protein *c* and *d*; Fig. S8†). These spots showed the lowest ratio for Cy5 to Cy3 signals in the 47 °C gel, suggesting that significant thermal destabilization occurred (Fig. 3d). Spots with the highest Cy5/Cy3 signal ratios were ruled out because they were also red color in the unheated gel, and thus their signal differences were not caused by thermal stability shift, but rather by PTM. Both spot *c* and *d* showed similar temperature-dependent thermal destabilization patterns, and the melting curve of protein *d* is presented in Fig. 3e. Mass spectrometry revealed the protein identity of both spot *c* and *d* as PKCα, the known target of bryostatin 1 (Table S1†). The thermal destabilization of PKCα by bryostatin 1 was confirmed by CETSA (Fig. 3f). Additionally, an isothermal dose–response curve at 48 °C revealed dose-dependent thermal destabilization of PKCα, supporting our observations (Fig. 3g). We performed CETSA for other PKC isozymes because bryostatin 1 is known to bind to other PKC isozymes.^16^ PKCβII and PKCθ showed thermal destabilization, while PKCβI, PKCδ, and PKCε showed no significant thermal stability shifts, and the anticipated bands of PKCγ, PKCμ, and PKCζ were not detectable (Fig. S5b and S9†).

### Identification of a novel target protein of hordenine using TS-FITGE

**Fig. 4:**
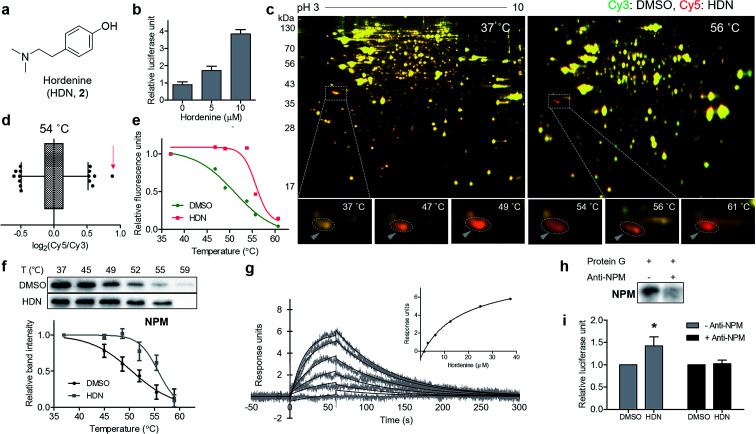
Identification of the novel target protein of hordenine. (a) Chemical structure of hordenine (*N*,*N*-dimethyltyramine, HDN). (b) Upregulational effect of HDN on *in vitro* translation assay measured by luciferase reporter gene product. Data are presented as the mean ± s.e.m. (*n* = 2). (c) Representative images from the TS-FITGE experiment. Images of Cy3 channel (green, proteome treated with DMSO) and Cy5 channel (red, proteome treated with HDN) were overlaid. (d) Box plot showing distribution of Cy5/Cy3 fluorescence ratio for each spot in the 54 °C gel. The whiskers indicate 1–99 percentiles. The spot pointed with a triangle in (c) was indicated by a red arrow. (e) Melting curves showing denaturation pattern of the spot indicated in (d). (f) Immunoblot from cellular thermal shift assay with the nucleophosmin (NPM) antibody. Data are presented as the mean ± s.e.m. (*n* = 4). (g) Sensorgrams from surface plasmon resonance assay showing the binding kinetics of HDN (1.56–37.5 μM) to immobilized NPM. Inset shows the steady-state response against various concentrations of HDN. (h) Immunoblot with NPM antibody showing depletion of NPM by anti-NPM bound protein G beads. (i) Decreased efficacy of HDN on translational upregulation after NPM depletion. Data are presented as the mean ± s.e.m. (*n* = 3), * *P* < 0.05 by paired student's *t*-test.

Finally, we applied our TS-FITGE method to a bioactive small natural product that has a simple chemical structure lacking room for chemical modification. Hordenine, *N*,*N*-dimethyltyramine (Fig. 4a), is an alkaloid found in several plants, particularly sprouting barley.^17^ Hordenine has several bioactivities such as the stimulation of gastrin release in rats,^18^ the inhibition of norepinephrine uptake in isolated vasa deferentia,^19^ and the increases in respiratory and heart rates when intravenously injected into horses.^20^ From screening of small-molecule libraries to identify compounds affecting *in vitro* protein translation, hordenine was found to increase the translation of the luciferase reporter gene (Fig. 4b). However, we found no previous reports examining the biological activity of hordenine and its binding partners related to protein translation. We adopted HEK293T cell line from which we would identify target proteins of hordenine because we assumed that protein translation would be conserved across most cell types. A reddish spot was detected from the TS-FITGE experiment (Fig. 4c and S10†), and that spot showed the highest Cy5/Cy3 ratio in the 54 °C gel (Fig. 4d). This spot was quantified over a range of temperatures to plot the sigmoidal melting curve (Fig. 4e), and the gel spot was excised for LC-MS/MS analysis to obtain a list of target candidates (Table S1†). To determine the binding partner, we performed CETSA of nucleophosmin (NPM), elongation factor 1-delta (EEF1d), protein SET (SET), and 40S ribosomal protein SA (RPSA), which are related to protein translation (Fig. 4f and S11a†). NPM showed significant thermal stabilization compared to β-tubulin and GAPDH, while the other candidate proteins did not. Dose-dependent thermal stabilization of NPM by hordenine was confirmed by the isothermal dose–response plot at 53 °C with an EC_50_ value of 7.6 μM (Fig. S11b†). To confirm the direct binding of hordenine to NPM, we conducted surface plasmon resonance analysis, which clearly showed a concentration-dependent response of the conventional one-to-one binding pattern with a *K*_D_ value of 13.6 μM (Fig. 4g).

To determine whether the upregulation of *in vitro* protein translation was caused by the functional modulation of NPM by hordenine, we conducted the functional validation *via* a loss-of-function study by depleting NPM from the *in vitro* translation system. Briefly, NPM antibody bound to protein G on agarose beads was incubated with the *in vitro* translation system to scavenge NPM. After removing the agarose beads, the remaining NPM in the solution was reduced, as observed in the western blotting results (Fig. 4h). The original upregulating efficacy of hordenine on luciferase gene translation was significantly diminished by 72.7% after NPM depletion (Fig. 4i), validating that hordenine upregulated *in vitro* protein translation of the luciferase reporter gene by directly regulating NPM.

### Pros and cons of target identification using TS-FITGE

Although gel-free proteomics has been favored as a result of rapidly advancing LC-MS technique, 2-DE remains an effective proteomic approach because of its affordability, robustness, and resolution.^21,22^ Our TS-FITGE method efficiently defined protein spots with shifted thermal stability simply by distinguishing red or green (*i.e.*, fluorescence difference between Cy3 and Cy5 signals) spots from other yellow spots *via* intra-gel image analysis. Subsequent quantification of the relative abundance of individual protein spots at various temperatures by automated inter-gel image analysis enabled melting curve plotting to confirm whether the spots exhibited sigmoidal thermal denaturation or false-positive signals. Therefore, the TS-FITGE method can be used to identify the shift in thermal stability among various temperatures, even when the melting curve slope is steep and the *T*_m_ shift is marginal (Fig. S12†). This longitudinal comparison criteria (deviation along the *y*-axis between two melting curves) rationalize the compatibility of TS-FITGE with the remarkable work of quantitative MS-based thermal proteome profiling (TPP),^23,24^ which used transverse comparison criteria (melting curve shift along the *x*-axis); the TPP method measured relative peptide abundance across 10 temperature points by using the lowest temperature condition as a reference. Consequently, *T*_m_ was calculated within vehicle- and drug-treated groups separately, and a difference in *T*_m_ between two groups was the main readout in TPP.

Notably, as shown for bryostatin 1, the TS-FITGE method can easily distinguish colored protein spots caused by thermal shift from those by PTM outcomes. Red or green spots appearing in the unheated gel were likely caused by PTMs upon treatment with drugs, not by thermal stability shifts. Given the short duration of drug treatment (*i.e.*, approximately 30 min), the colored spots in the unheated gel may not have been caused by changes in expressional levels. For instance, a red spot and a green spot appeared next to each other horizontally following treatment with bryostatin 1 at the 37 °C gel (Fig. S6b,† protein *f* and *g*) were identified as cytoplasmic dynein 1 intermediate chain 2 (DC1I2) (Table S1†). This indicates that bryostatin 1 induced a PTM of DC1I2 and shifted its isoelectric point, which is consistent with previous reports showing that DC1I2 is phosphorylated in a PKC-dependent manner.^25–27^ Similarly, horizontal colored protein spots (Fig. S6b,† protein *h*, *i*, and *j*) were all identified as heterogeneous nuclear ribonucleoprotein K (hnRNP K) (Table S1†). The interaction of hnRNP with PKC and subsequent phosphorylation of hnRNP have been well documented in various studies.^28–31^ Thus, the TS-FITGE method can not only identify the target proteins of bioactive small molecules but also reveal additional information about their downstream signaling pathways, which is useful for understanding the mechanism-of-action of bioactive small molecules in the cellular environment.

However, some proteins, especially transmembrane proteins or large protein complexes, may not show thermal stability shift upon drug engagement, and these proteins are not applicable for any thermal shift-based label-free target ID methods including TPP, CETSA, and TS-FITGE. In addition, identification of target proteins with low abundance in 2-DE using our method remains to be a challenge. In the case of bryostatin 1, although PKCβII and PKCθ showed thermal destabilization according to CETSA, they were not identified as target proteins by the TS-FITGE method, likely because of their low abundance and the limitations of in-gel fluorescence detection. Therefore, identifying low-abundant target proteins still remains an urgent problem in the field of label-free target identification, as suggested by Reinhard *et al.*^32^

## Conclusions

In summary, we established a proteome-wide label-free method for target ID using thermal stability shift combined with our original FITGE technique. This label-free target ID method is useful for bioactive natural products for which it is difficult to synthesize their target ID probes because of structural complexity or the lack of modification sites. In the TS-FITGE method, fluorescence differences between relative red and green signals by automated intra-gel image analysis reflected changes in a protein's thermal stability upon drug engagement. Additionally, the melting curve for each protein was plotted to confirm the temperature-dependent thermal denaturation and measure *T*_m_ shifting through quantitative inter-gel image analysis. Using this TS-FITGE method, both thermal stabilization and destabilization of target proteins were detected, and membrane-anchored proteins were identified by the addition of mild detergent to the lysis buffer. Furthermore, we identified nucleophosmin as a novel target protein of hordenine, a natural product upregulator of *in vitro* protein translation. These findings suggest that our TS-FITGE method can expand the accessibility of label-free target ID for the development of first-in-class therapeutics or research tools in biomedical research.

## Supplementary Material

SC-008-C6SC03238A-s001
