# Supplementary material for: Label-free target identification using in-gel fluorescence difference via thermal stability shift
Source: Chem Sci. 2016 Sep 22;8(2):1127–33. doi: 10.1039/c6sc03238a (PMC5369521; doi:10.1039/c6sc03238a)
Supplement: SC-008-C6SC03238A-s001 [file SC-008-C6SC03238A-s001.pdf]

Electronic Supplementary Material (ESI) for Chemical Science.
This journal is © The Royal Society of Chemistry 2016

< Supplementary Information >

Label-Free Target Identification Using In-Gel Fluorescence Difference via Thermal Stability Shift

*Hankum Park, Jaeyoung Ha, Ja Young Koo, Jongmin Park, and Seung Bum Park**

Table of contents

1. Supplementary Figures

   Figure S1-S14 — S2–S14

2. Supplementary Table

   Table S1 — S15

3. Methods — S16–S18
4. Chemical synthesis — S19–S27
5. Supplementary references — S27
6. Appendices: NMR spectra — S28–38

1. Supplementary Figures

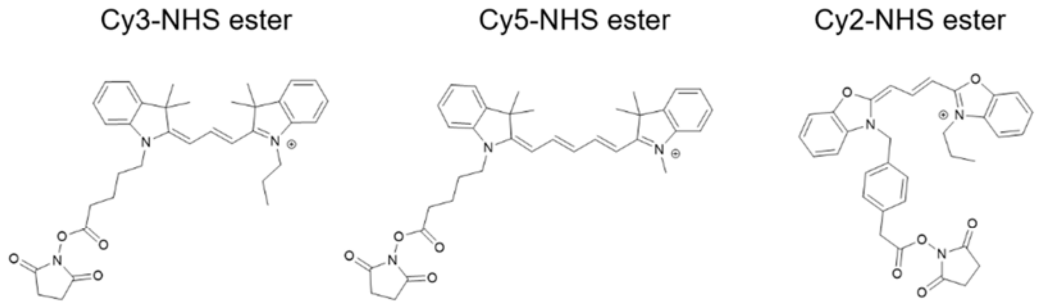

Formula	$C_{35}H_{42}N_3O_4^+$	$C_{35}H_{40}N_3O_4^+$	$C_{33}H_{30}N_3O_6^+$
MW	568.74	566.72	564.62

Fig. S1 Chemical structure, formula, and molecular weight of three fluorescent dyes.

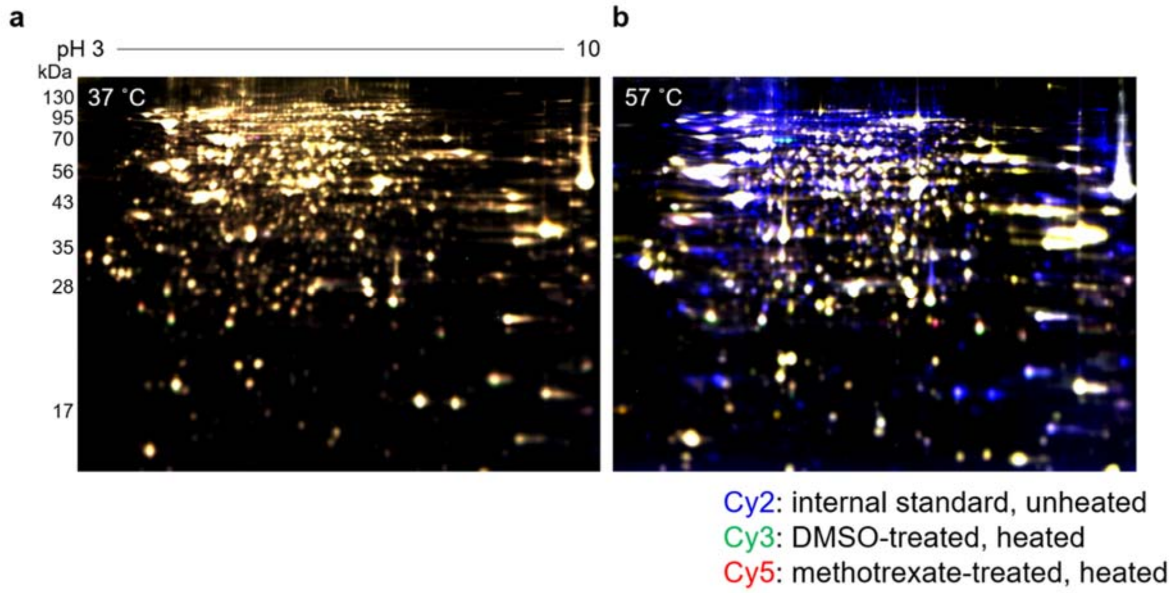

Fig. S2 Representative images showing the same mobility of proteomes conjugated with three different dyes. (a) Cy2, Cy3, and Cy5 signals were overlapped at the 37 °C gel, and most spots appeared as white color. (b) At 57 °C, thermally unstable proteins were precipitated and removed from soluble fraction. Therefore, those spots were shown as blue color from unheated internal standard which was conjugated with Cy2 dye.

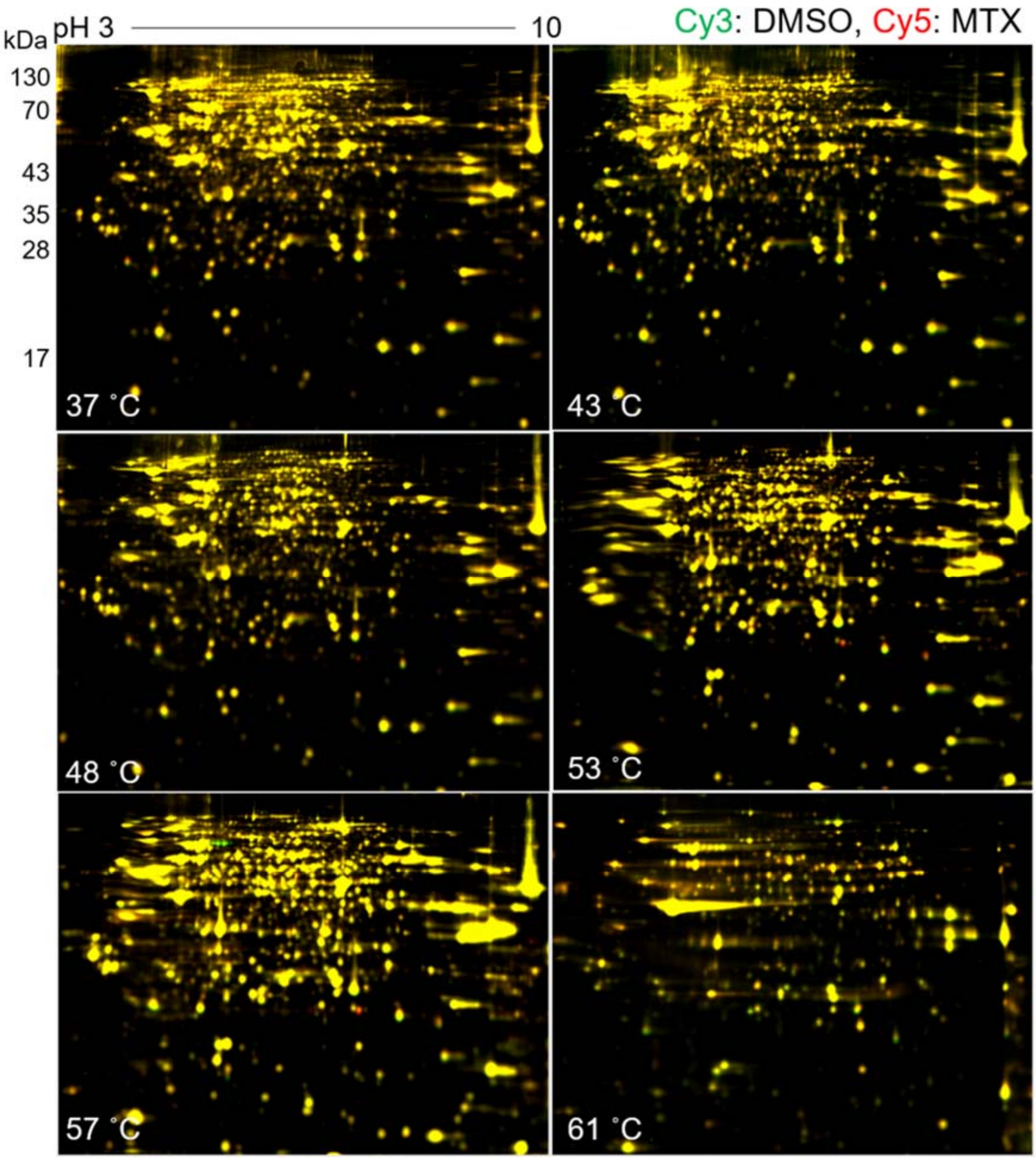

Fig. S3 Whole gel images from TS-FITGE experiments with methotrexate (MTX).

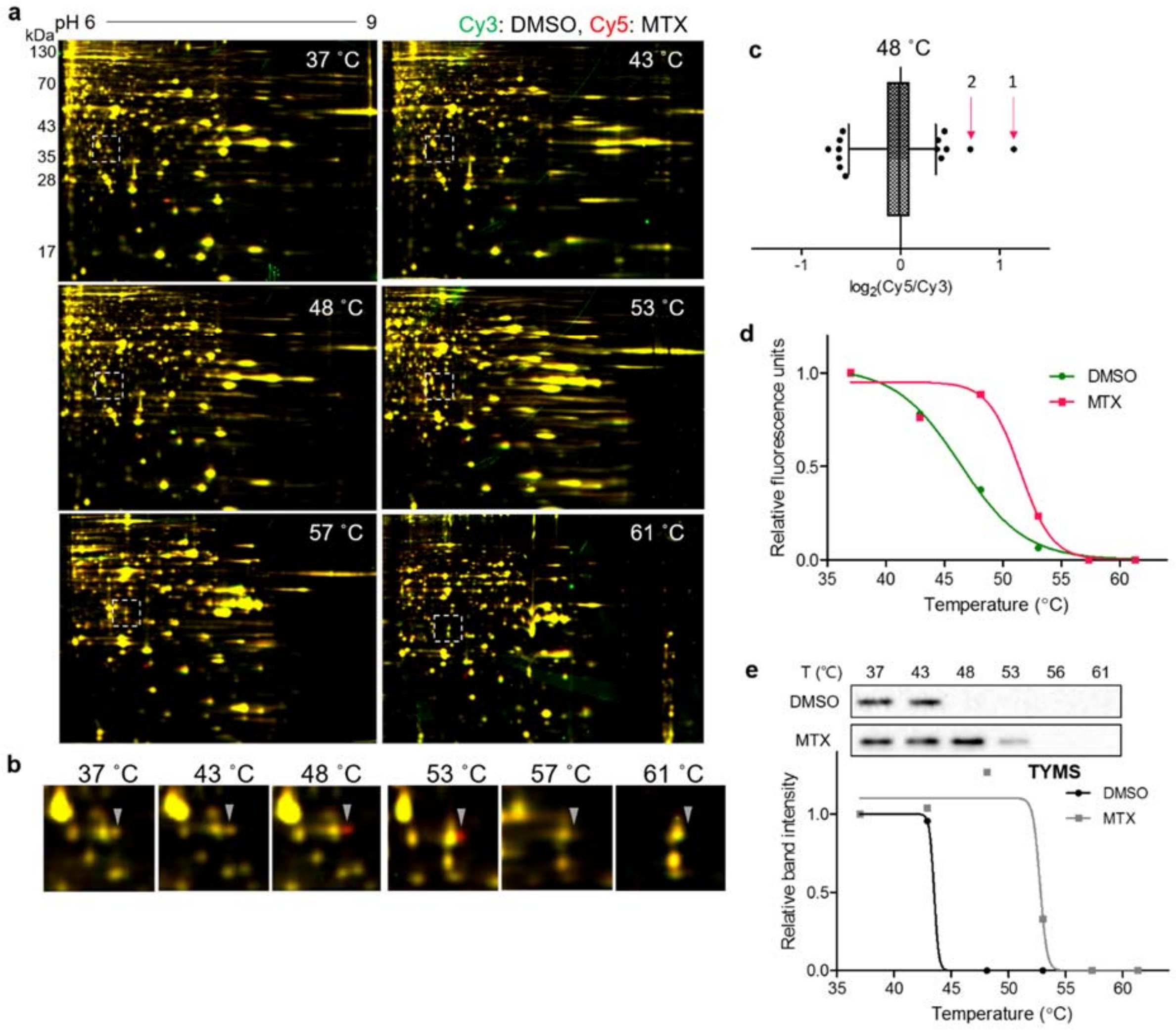

Fig. S4 Identification of thymidylate synthase as another target protein of methotrexate (MTX). (a) Whole gel images of from TS-FITGE experiments with MTX. Strip gels of pH gradient 6 to 9 were used for better resolution in basic pH range. (b) Enlarged images of the region indicated by white box in (a). (c) Box plot showing the distribution of Cy5/Cy3 signal ratio for each spot in the 48 °C gel. The whiskers indicate 1–99 percentiles. The spot pointed by a triangle in (b) is indicated by a red arrow **1**. Red arrow **2** is the spot of dihydrofolate reductase (DHFR). (d) Melting curves showing the denaturation pattern of the arrowed spot **1** in (c). (e) Immunoblot from cellular thermal shift assay with thymidylate synthase (TYMS) antibody.

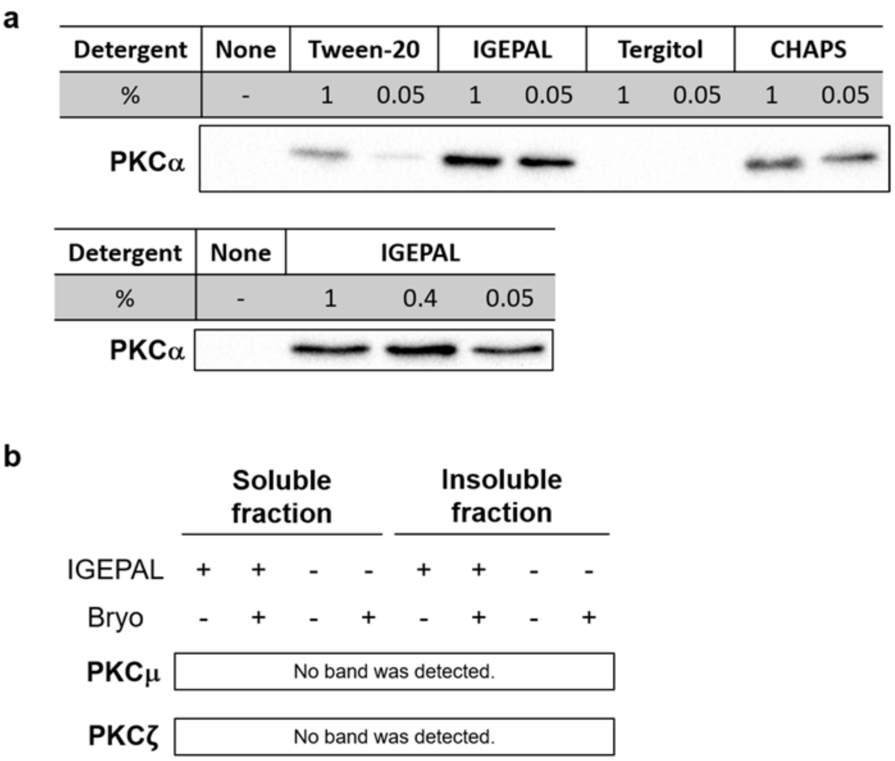

Fig. S5 The effect of detergent on solubilization of PKCs. (a) Optimization of the detergent condition in the lysis buffer using PKCα as a representative of PKCs for the initial screening. The amount of PKCα in the soluble fraction upon 1-h treatment with bryostatin 1 was measured by western blot. IGEPAL CA-630 effectively solubilized PKCα. 0.4% (v/v) was better than 0.05% and similar to 1% for PKCα solubilization. (b) Translocation of PKCμ and PKCζ upon 1-h treatment with bryostatin 1 and the effect of 0.4% of IGEPAL CA-630 on their solubilization.

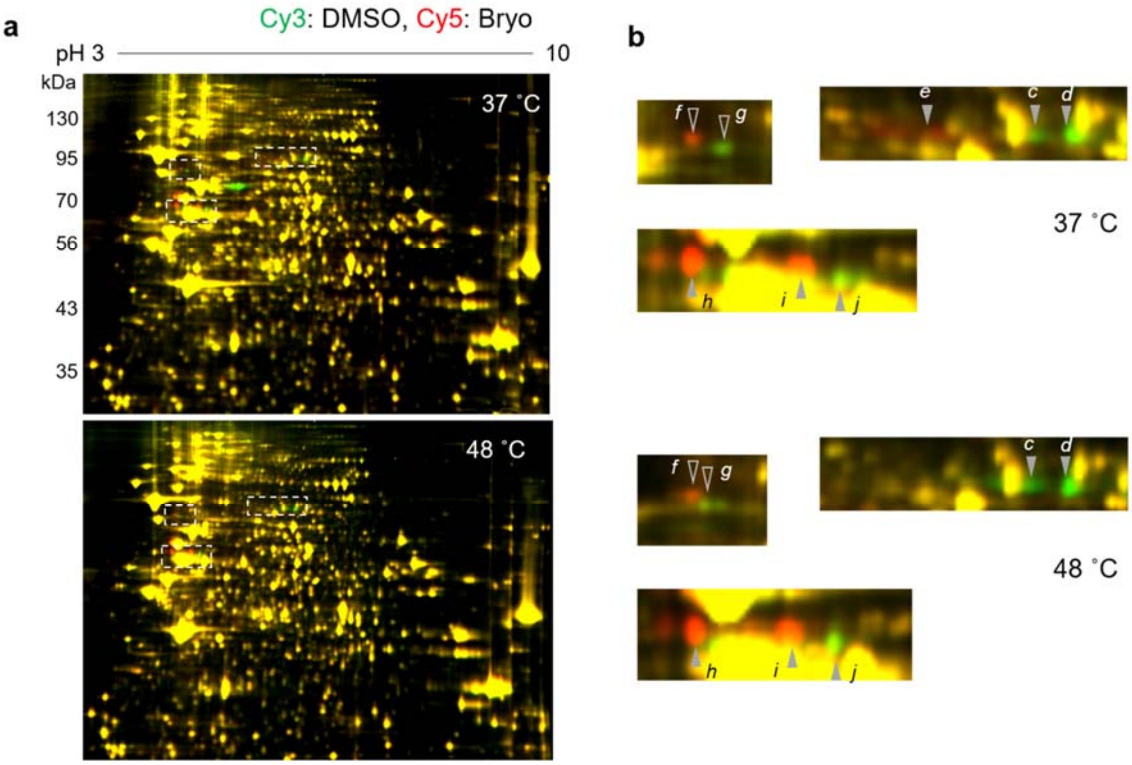

Fig. S6 Appearance of red and green spots upon treatment of bryostatin 1 without heat denaturation. (a) Representative images from the TS-FITGE experiment upon treatment with bryostatin 1 for 75 min. (b) Enlarged images of the regions indicated by white boxes in (a).

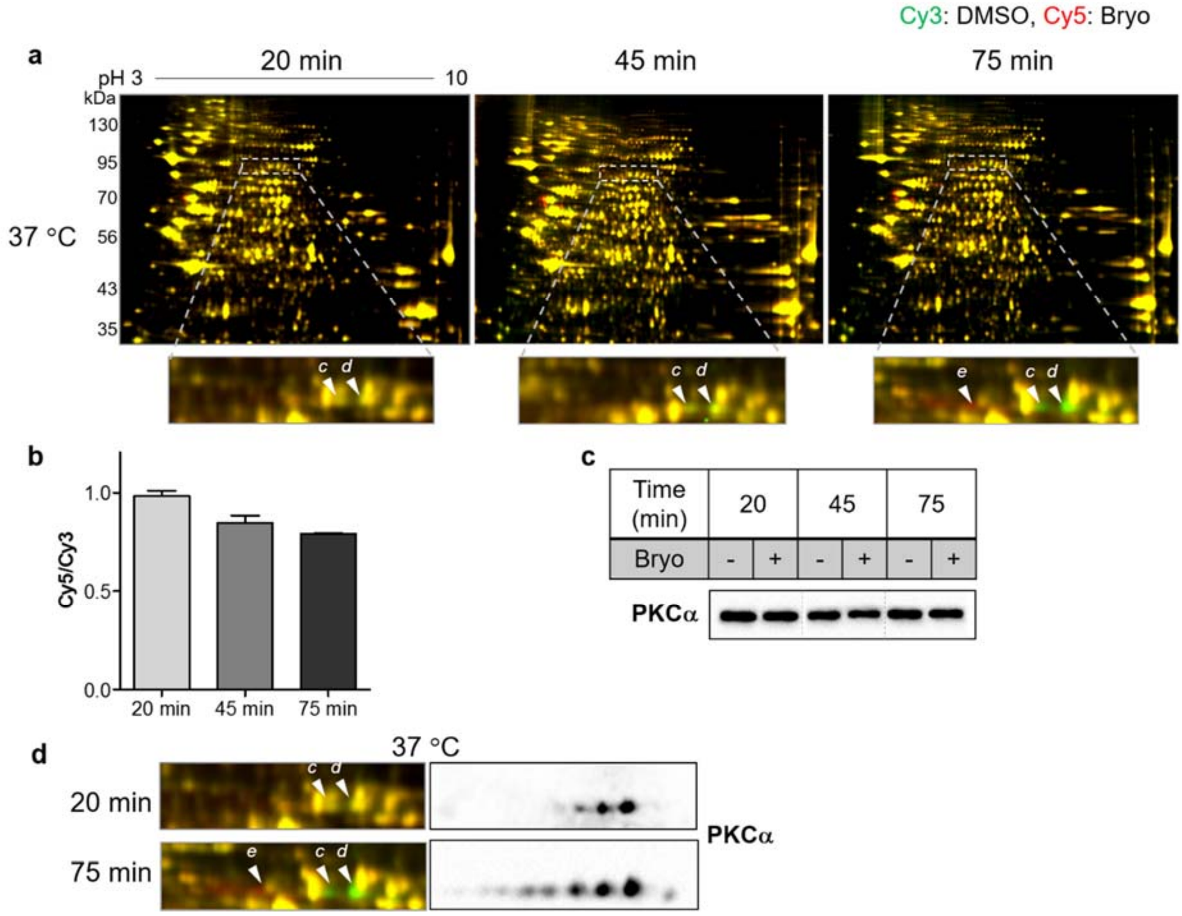

Fig. S7 Effect of the treatment time on protein spot shift due to post-translational modification. (a) 2D gel analysis of color change upon treatment of bryostatin 1 without thermal denaturation. As the treatment time decreased, the red spots including *e* disappeared, and the green spots *c* and *d* became yellow. (b) The quantitative ratio of Cy5 to Cy3 signals of the protein spot *d*. (c) Immunoblot with PKCα antibody on a 1D gel to compare the total amount of PKCα. The total amounts of PKCα were identical regradless of treatment time at 37 °C. Althouth the lanes look discontinuous, they are cropped from the single membrane with same contrast. (d) Immunoblot with PKCα antibody on 2D gels. Treatment for 75 min induced the appearance of additional PKCα spots, which indicates the change in isoelectric point due to post-translational modification. The additional spots appeared as red color, and the remaining spots became green on the fluorescence image.

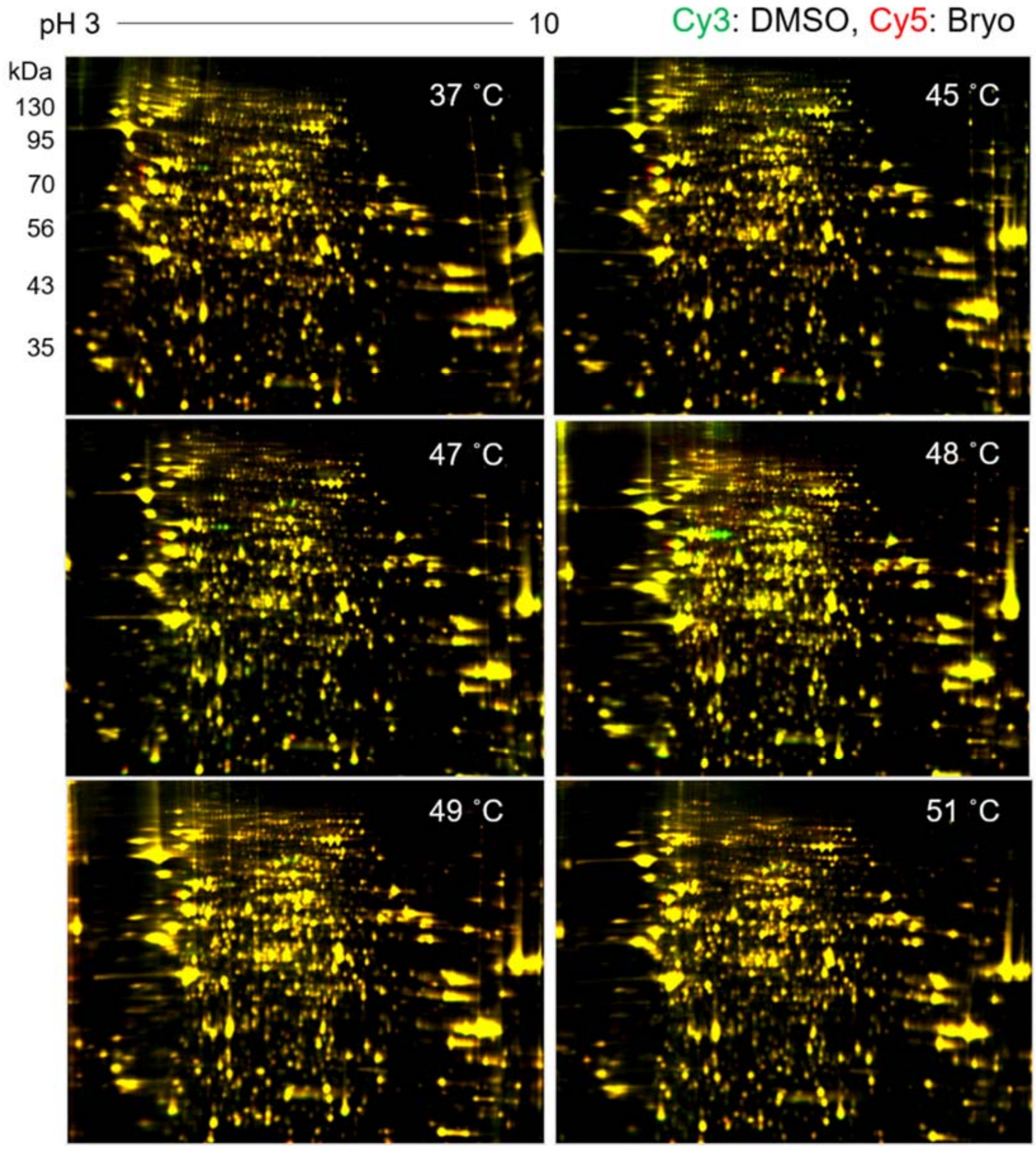

Fig. S8 Whole gel images from TS-FITGE experiments upon treatment with bryostatin 1 for 20 min.

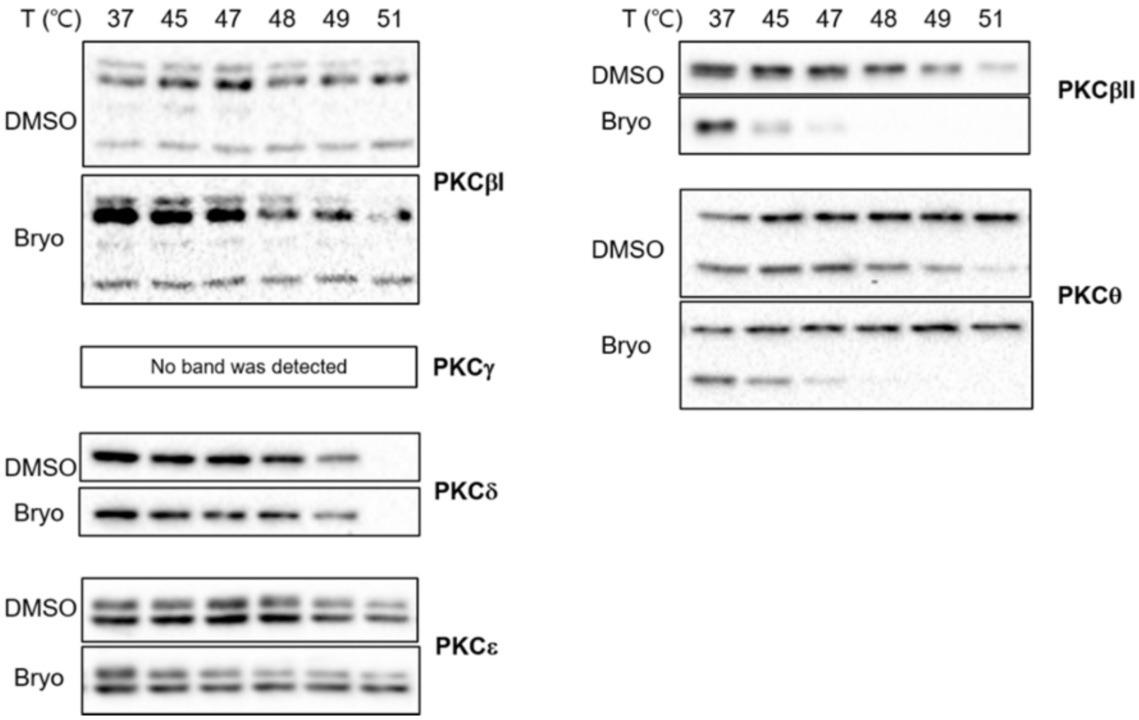

Fig. S9 CETSA analysis against PKC isozymes. PKCβII and PKCθ showed thermal destabilization, while the others did not.

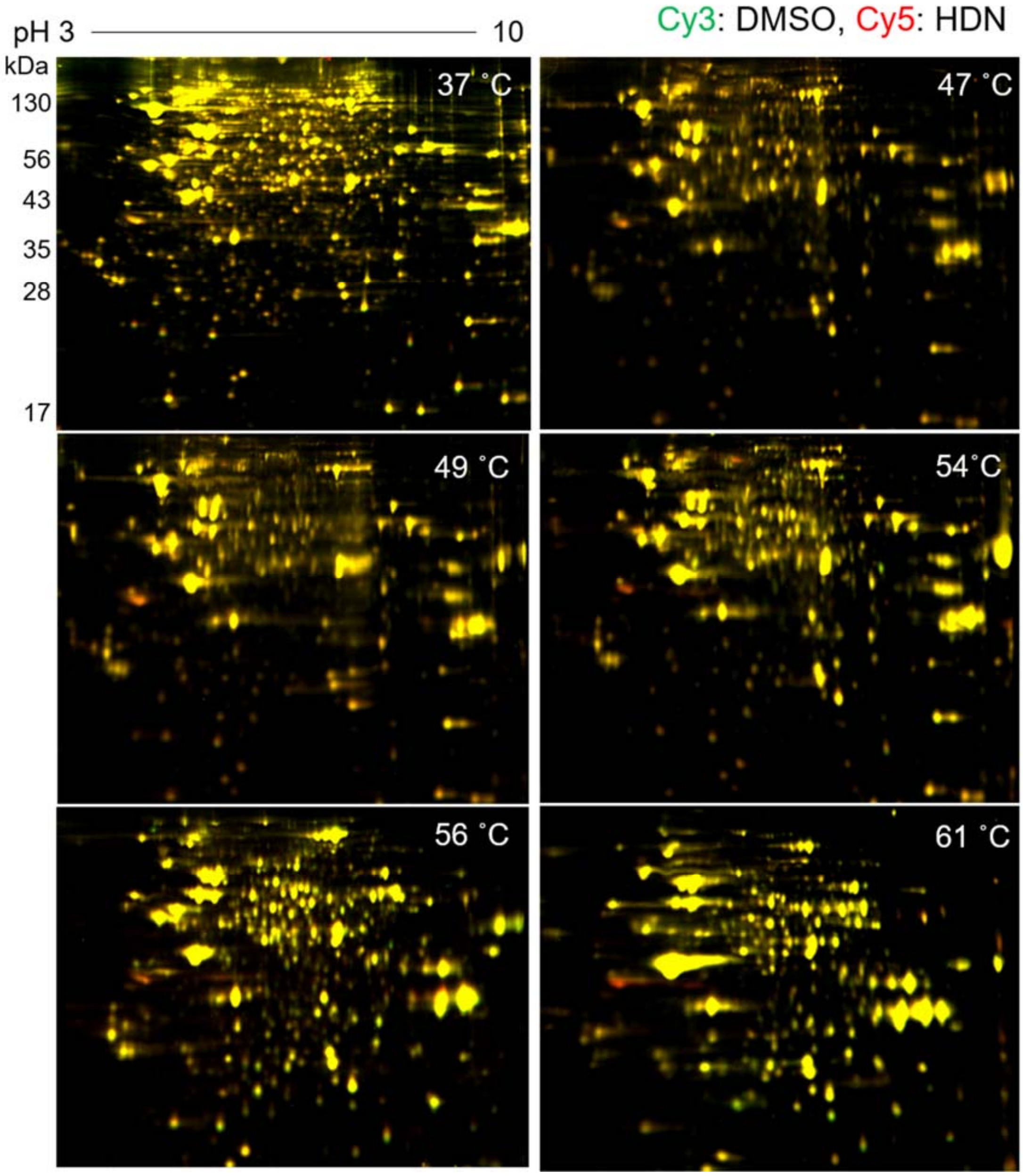

Fig. S10 Whole gel images from TS-FITGE experiments upon treatment with hordenine (HDN).

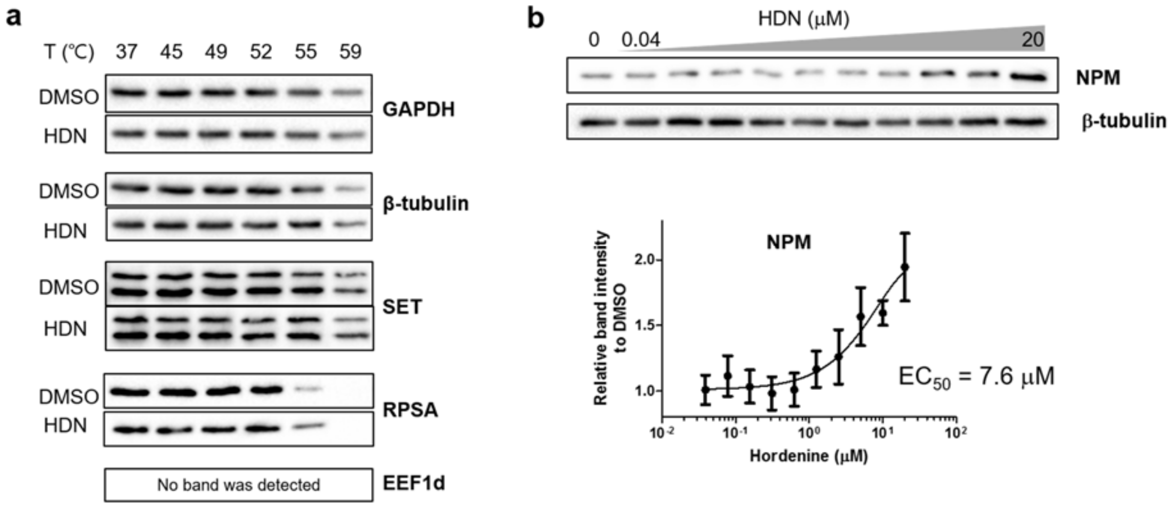

Fig. S11 Immunoblot analysis to test whether the target protein showed thermal stabilization. (a) Cellular thermal shift assay against SET, RPSA, and EEF1d with control protein, GAPDH and β-tubulin. (b) Isothermal dose-response analysis of NPM and β-tubulin at 53 °C. NPM protein showed a dose-dependent thermal stabilization by HDN.

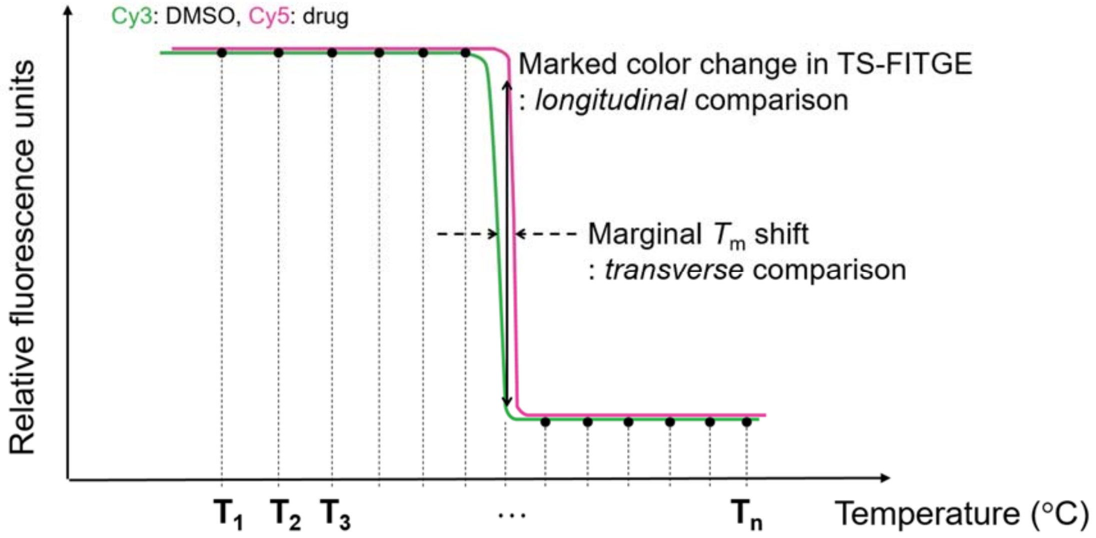

Fig. S12 Description of the analysis procedure in the TS-FITGE. As the fluorescence difference at each temperature is directly compared in TS-FITGE, marked color change can be detected at a certain temperature even if T_m shift is marginal. The certain temperaure point can be captured by simply narrowing the temperature intervals via additional number of gels.

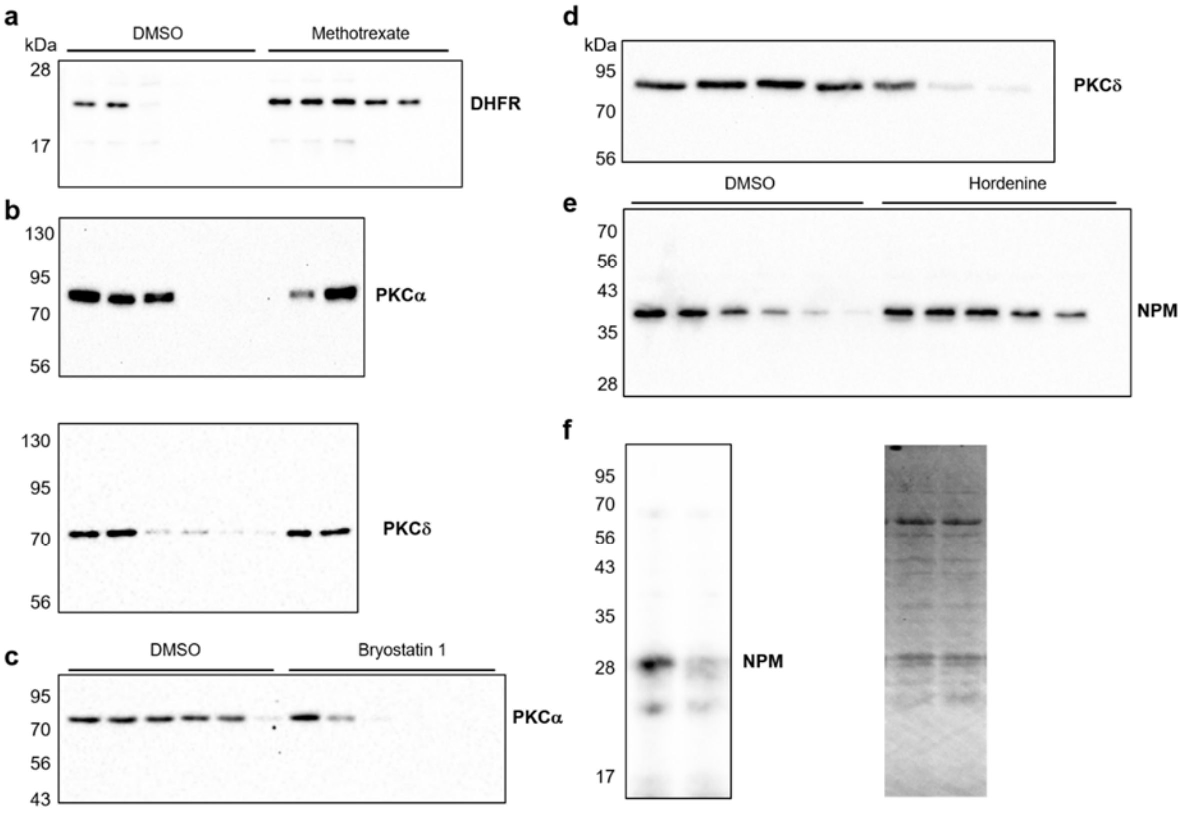

Fig. S13 Uncropped immunoblot images of (a) Fig. 2d, (b) Fig. 3b, (c) Fig. 3f, (d) Fig. 3g, (e) Fig. 4f, and (f) Fig. 4h (Coomassie-stained image of the membrane is shown in the right hand side).

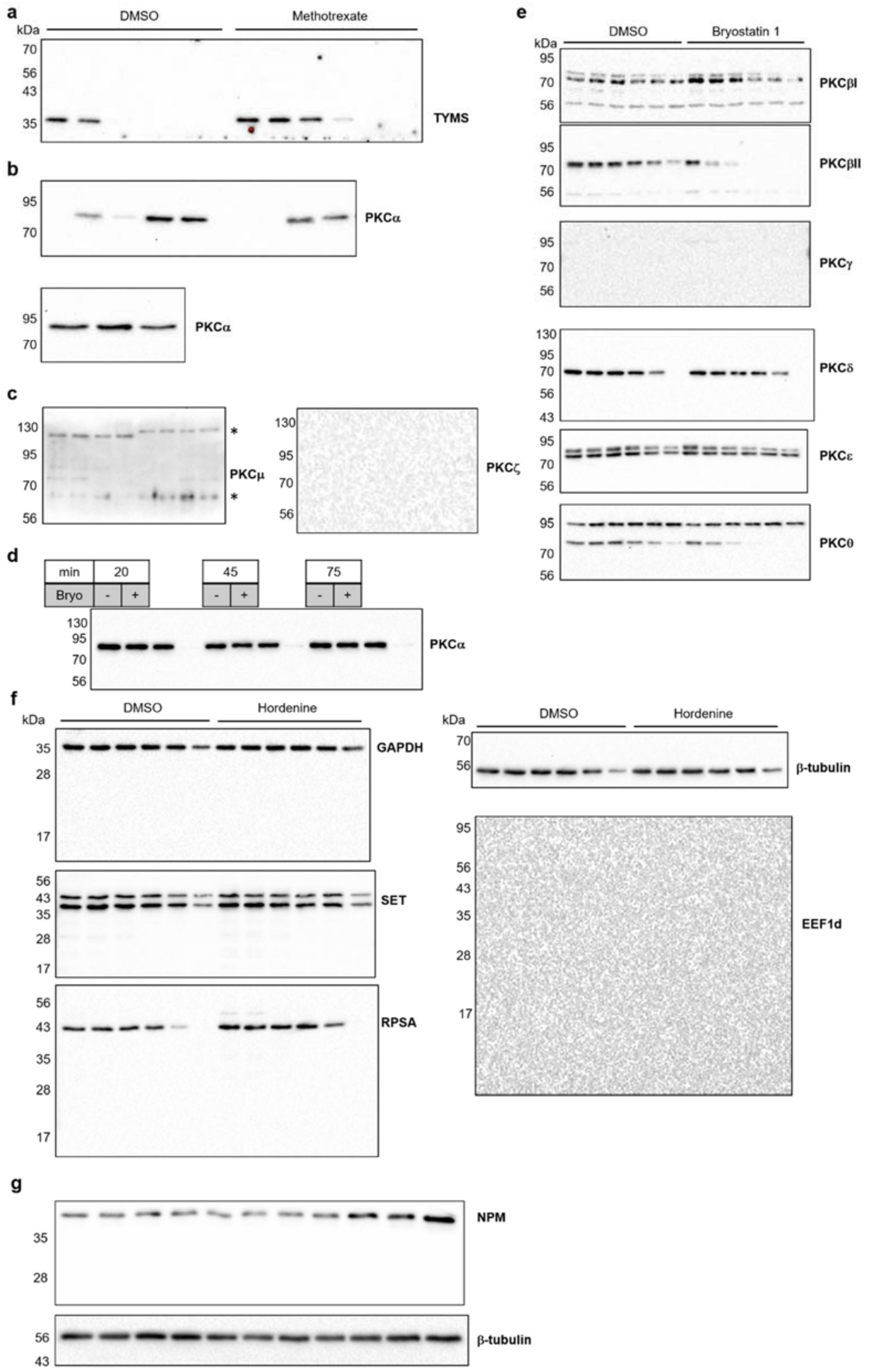

Fig. S14 Uncropped immunoblot images of (a) Fig. S4e, (b) Fig. S5a, (c) Fig. S5b (asterisks indicate unspecific bands), (d) Fig. S7c, (e) Fig. S9, (f) Fig. S11a, and (g) Fig. S11b.

2. Supplementary Table

Table S1. Protein identification of the spots in 2-DE by mass spectrometry. Proteins having Mascot score greater than 100 were listed as they showed reproducible results.

Compound	Protein spot	Match to	Molecular Weight	Mascot Score	Queries matched	Sequence Coverage (%)	Protein
Methotrexate	Fig.2a	DYR_HUMAN	21439	533	79	82	Dihydrofolate reductase
	Supplementary Fig.4b	TYSY_HUMAN	35693	527	19	37	Thymidylate synthase
		PRPS1_HUMAN	34812	224	11	38	Ribose-phosphate pyrophosphokinase I
Bryostatin 1	Fig.3c, *a*	ELMO1_HUMAN	83776	210	19	21	Engulfment and cell motility protein 1
	Fig.3c, *b*	ELMO1_HUMAN	83776	287	27	30	Engulfment and cell motility protein 1
	Fig.3c, *c*	MCM7_HUMAN	81257	615	29	39	DNA replication licensing factor MCM7
		KPCA_HUMAN	76714	220	10	18	Protein kinase C alpha type
		SYTC_HUMAN	83382	183	12	20	Threonyl-tRNA synthetase, cytoplasmic
	Fig.3c, *d*	KPCA_HUMAN	76714	307	18	28	Protein kinase C alpha type
		SYTC_HUMAN	83382	135	11	18	Threonyl-tRNA synthetase, cytoplasmic
	Supplementary Fig.6b, *e*	MCM7_HUMAN	81257	276	10	18	DNA replication licensing factor MCM7
		KPCA_HUMAN	76714	132	7	12	Protein kinase C alpha type
		EFG1_HUMAN	83418	108	5	8	Elongation factor G 1, mitochondrial precursor
	Supplementary Fig.6b, *f*	DC1I2_HUMAN	71412	395	15	32	Cytoplasmic dynein 1 intermediate chain 2
		GRP78_HUMAN	72288	119	9	16	78 kDa glucose-regulated protein precursor
	Supplementary Fig.6b, *g*	DC1I2_HUMAN	71412	489	12	26	Cytoplasmic dynein 1 intermediate chain 2
		GRP78_HUMAN	7228	195	13	24	78 kDa glucose-regulated protein precursor
	Supplementary Fig.6b, *h*	HNRPK_HUMAN	50944	488	25	57	Heterogeneous nuclear ribonucleoprotein K
	Supplementary Fig.6b, *i*	HNRPK_HUMAN	50944	405	23	40	Heterogeneous nuclear ribonucleoprotein K
		PGTA_HUMAN	65030	144	11	22	Geranylgeranyl transferase type-2 alpha subunit
		HDAC1_HUMAN	55068	126	4	12	Histone deacetylase 1
	Supplementary Fig.6b, *j*	HNRPK_HUMAN	50944	519	25	52	Heterogeneous nuclear ribonucleoprotein K
		PGTA_HUMAN	65030	399	15	29	Geranylgeranyl transferase type-2 alpha subunit
		CH60_HUMAN	61016	354	17	30	60 kDa heat shock protein, mitochondrial precursor
		TCPQ_HUMAN	59583	173	10	21	T-complex protein 1 subunit theta
		TCPE_HUMAN	59633	153	14	33	T-complex protein 1 subunit epsilon
Hordenine	Fig.4c	NPM_HUMAN	32555	5562	175	87	Nucleophosmin
		EF1D_HUMAN	31103	1847	40	77	Elongation factor 1-delta
		SET_HUMAN	33469	1039	47	48	Protein SET
		TTC1_HUMAN	33505	888	45	75	Tetratricopeptide repeat protein 1
		RSSA_HUMAN	32833	347	11	29	40S ribosomal protein SA
		HS90B_HUMAN	83212	295	11	14	Heat shock protein HSP 90-beta
		HDGF_HUMAN	26772	265	9	51	Hepatoma-derived growth factor
		VA0D_HUMAN	40303	223	12	28	Vacuolar ATP synthase subunit d

3. Methods

Chemicals and reagents. All chemicals including methotrexate, bryostatin 1, hordenine, and octylphenoxy poly(ethyleneoxy)ethanol branched (IGEPAL CA-630) were purchased from Sigma-Aldrich. Cell culture reagents including media, fetal bovine serum (FBS), and antibiotic-antimycotic solution were from Gibco [Life technologies]. Dulbecco's phosphate-buffered saline was purchased from Welgene. Complete protease inhibitor cocktail (EDTA-free) was from Roche.

Cell culture. HEK293T and Jurkat clone E6-1 cells were purchased from Korea cell line bank. HEK293T cells were cultured in DMEM supplemented with 10% (v/v) FBS and 1% (v/v) antibiotic-antimycotic solution. Jurkat cells were cultured in RPMI 1640 media supplemented with 10% (v/v) FBS and 1% (v/v) antibiotic-antimycotic solution. Cells were maintained at 37 °C in humidified 5% CO_2 incubator.

Preparation of cell extract. Jurkat cell was used for bryostatin 1[1] and HEK293T cell for methotrexate[2] and hordenine. HEK293T cells were harvested with trypsin/EDTA solution and resuspended in a conical tube before compound treatment. Methotrexate was incubated with HEK293T cells at 10 μM for 3 h, bryostatin 1 with Jurkat cells at 50 nM for various times, and hordenine with HEK293T cells at 20 μM for 3 h. After the compound treatment, cells were aliquoted, heated to a range of temperature for 3 min, and cooled to 25 °C for 3 min. The heated cells were harvested, washed with PBS, and resuspended with lysis buffer (PBS supplemented with protease inhibitor cocktail). The cell suspension was freeze-thawed three times with liquid nitrogen for cell lysis. Soluble fraction was separated from the cell lysate following centrifugation at 20,000g for 20 min at 4 °C. Procedures for isothermal dose-response (ITDR) analysis was identical with the above mentioned procedure except that cells were treated with various compound concentrations and heated at a fixed temperature.

Western blot. The soluble fraction was mixed with 5× Laemmli buffer followed by heating at 95 °C for 5 min. Proteins were separated by SDS-PAGE and transferred to PVDF membranes. The membranes were blocked by 2% BSA in tris-buffered saline with Tween-20 (TBST) for 1 h and incubated with primary antibodies—anti-DHFR [ab133546, abcam], anti-TYMS [3766, Cell signaling], anti-PKCα [2056, Cell signaling], anti-PKCβI [ab195039, abcam], anti-PKCβII [ab32026, abcam], anti-PKCγ [ab131222, abcam], anti-PKCδ [9616, Cell signaling], anti-PKCε [ab124806, abcam], anti-PKCμ [2052, Cell signaling], anti-PKCθ [ab110728, abcam], anti-PKCζ [9368, Cell signaling], anti-NPM [ab52644, abcam], anti-RPSA [ab133645, abcam], anti-SET [ab176567, abcam], anti-EEF1D [ab88868, abcam], anti-GAPDH [2118, Cell signaling], and anti-β-tubulin [2146, Cell signaling]—overnight at 4 °C. The membrane then washed with TBST and incubated with secondary antibodies—anti-rabbit IgG, HRP-

linked [7074, Cell signaling]—for 1 h at room temperature. The membranes were developed with Amersham ECL prime western blotting detection reagent [GE Healthcare], and detected by ChemiDoc [Bio-Rad]. The relative band intensities to 37 °C was calculated within drug-treated group and control group each for the CETSA curves. For ITDR curves, the relative band intensities to DMSO group was calculated. Data analysis and sigmoidal curve fitting were done by GraphPad Prism 5.

2D gel electrophoresis. Protein concentration of the soluble fraction of cell extract was quantified with Bio-Rad protein assay. Acetone was added to 50 μg of the protein and incubated at –20 °C for 1 h. The mixture was centrifuged at 20,000g for 20 min at 4 °C. Supernatant was discarded, and the pellet was washed with cold acetone twice. The residual pellet was sonicated for resuspension with 10 μl of conjugation buffer (30 mM Tris-HCl (pH 8.6), 2 M thiourea, 7 M urea, 4% CHAPS (w/v)) by sonication. 1 μl of 0.4 mM Cy3-NHS or Cy5-NHS were mixed to the resuspended proteins and incubated at 4 °C for 50 min. 320 μg of unheated sample for internal standard was precipitated, resuspended with 64 μl of the conjugation buffer, and mixed with 6.4 μl of Cy2-NHS. After the conjugation, cold acetone was added and incubated at –20 °C for 1 h. The mixture was centrifuged at 20,000g for 20 min at 4 °C. Supernatant was discarded, and the pellet was washed with cold acetone twice. Heated samples were sonicated for resuspension with 50 μl of rehydration buffer (7 M urea, 2 M thiourea, 2% CHAPS (w/v), 40 mM DTT, and 1% IPG buffer) and standard samples with 320 μl. 50 μl of the protein solution from both compound-treated group (conjugated with Cy5) and DMSO-treated group (conjugated with Cy3) were mixed; 50 μl of the unheated sample (conjugated with Cy2) was added for internal standard. Total 150 μg (50 μg for Cy2, Cy3, and Cy5 each) of proteins were loaded in a 24-cm Immmobiline Drystrip gel [GE Healthcare] which was rehydrated for 10 h, and isoelectric focusing was done by Ettan IPGphor 3 [GE Healthcare]. The proteins in the strip gel were separated by Ettan DALTsix electrophoresis system [GE Healthcare], the gel was scanned with Typhoon Trio [GE Healthcare].

Gel image analysis. Fluorescence signals from Cy2, Cy3, and Cy5 were quantified by DeCyder 2D software, ver. 7.2 [GE Healthcare]. Signal ratio values of Cy5 to Cy3 were normalized, so that the modal peak of the logarithm value of the ratio was set to be zero. The data were presented in a box-and-whisker diagram, and outliers were considered as thermally shifted spots. Used as internal standards for inter-gel analysis, Cy2 signal matched the location of each spot across all gels, and the relative amounts of Cy3 and Cy5 signals to Cy2 signal were calculated to plot melting curves. Data analysis and sigmoidal curve fitting were done by GraphPad Prism 5.

Mass spectrometry. The protein spots from silver-stained gel were excised and destained followed by in gel trypsin digestion. The extracts were evaporated in SpeedVac and then dissolved in 10% acetonitrile containing 0.1% formic acid. The resulting peptides were desalted with trap column (i.d.

180 μm × 20 mm, Symmetry C18) cartridge and separated on a C18 reversed-phase 75 μm i.d. × 200 mm analytical column (1.7 μm particle size, BEH130 C18, Waters) with integrated electrospray ionization PicoTip (±10 μm i.d., New Objective) using nanoAcquity UPLC-ESI-QTOF/MS [SYNAPT G2-Si HDMS, Waters]. The acquired data were converted to .pkl files with Protein Lynx Global Server and used to query the SwissProt database using MASCOT search.

***In vitro* protein translation**. TNT quick coupled transcription/translation system [Promega] was used for *in vitro* translation. TNT Quick Master Mix and T7 luciferase control DNA were mixed according to the manufacturer's protocol, and incubated with compounds at 30 °C for 1 h. After the addition of Luciferase assay reagent [Promega], luminescence from the control luciferase product was read by Synergy HT [BioTek] to measure the effect of compounds on protein translation.

Nucleophosmin depletion for functional validation. Protein G sepharose beads [Sigma] were washed with PBS and incubate with anti-NPM antibody at 4 °C for 2 h. Unbound antibodies were washed away. The anti-NPM bound Protein G beads were mixed with TNT Quick Master Mix and incubated at 4 °C for 2 h. The beads were removed by centrifugation, and the supernatant was incubated with T7 luciferase control DNA and 10 μM of hordenine at 30 °C for 1 h. Luminescence was read after the addition of Luciferase assay reagent.

Surface plasmon resonance assay. Binding kinetics were monitored by BIAcore T100 instrument [GE Healthcare]. Buffer condition of the human nucleophosmin full length protein [ab126664, abcam] was exchanged to PBS using 10K Amicon ultra centrifugal filter [Millipore]. The carboxyl group on CM5 chip surface was activated by injection of a mixture of NHS and EDC to both flow cells 1 and 2. Nucleophosmin protein (36 μg/mL) in acetate buffer (pH 4.0) was injected to the flow cell 2 for 550 sec with flow rate of 5 μl/min. Ethanolamine-HCl was injected to both flow cells 1 and 2 for quenching. Final immobilization level reached 9,400 RU and 12,800 RU for two independent experiments. For binding study, various concentration (1.56 μM to 37.5 μM) of hordenine was injected for 60 sec with flow rate of 30 μl/min and dissociated with injection of the running buffer (PBS supplemented with 0.005% P20, and 2% DMSO) for 400 seconds. Data were analyzed by BIAcore T100 Evaluation software [GE Healthcare], and the sensorgram was fitted to the 1:1 binding model.

3. Chemical synthesis

1. General Information for Synthesis

The ^{1}H and ^{13}C NMR spectra were recorded on a Varian DD2MR400 [Varian Assoc., USA], and chemical shifts were measured in ppm downfield from internal tetramethylsilane (TMS) standard. Multiplicity was indicated as follows: s (singlet); d (doublet); t (triplet); q (quartet); m (multiplet); dd (doublet of doublet); dt (doublet of triplet); td (triplet of doublet); qd (quartet of doublet); quind (quintet of doublet); tdd (triplet of doublet of doublet); brs (broad singlet), etc. Coupling constants were reported in Hz. Low resolution mass spectrometry (LRMS) analyses were performed with Finnigan MSQ Plus Surveyer HPLC/MS system [Thermo Electron Corp., USA] using electron spray ionization (ESI) and conducted at the national center for inter-university research facilities (NCRF) in Seoul National University using a mass spectrometer by direct injection for fast atomic bombardment (FAB). Analytical thin-layer chromatography (TLC) was performed using 0.25-mm silica-gel-coated Kiselgel 60 F_{254} plates, and the components were visualized by observation under UV light (254 and 365 nm) or by treating TLC plates with anisaldehyde or phosphomolybdic acid followed by thermal visualization. Silica gel 60 (0.040–0.063 mm) used in flash column chromatography was purchased from Merck. All reactions were conducted in oven-dried glassware under dry argon atmosphere, unless otherwise specified. All solvents and organic reagents were purchased from commercial venders [Sigma-Aldrich, TCI, Acros and Alfa Aesar] and used without further purification unless otherwise mentioned.

2. Preparation of Cy2, Cy3, Cy5 and their NHS-ester

Scheme 1. Synthesis of Cy2.

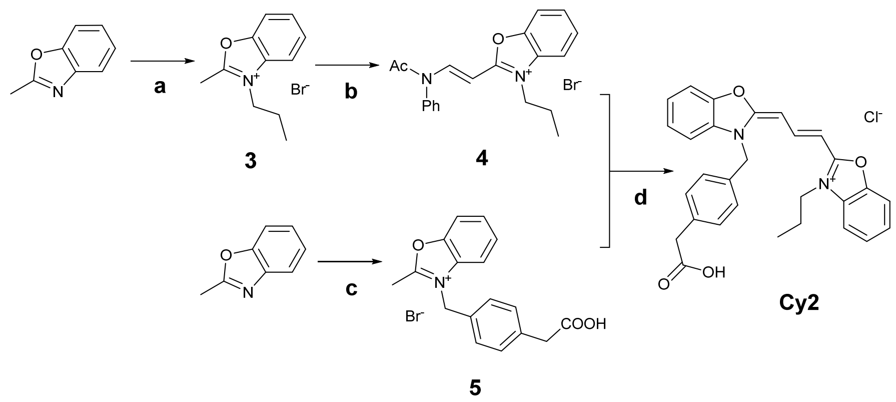

Reagents and conditions: (**a**) *n*-Propyl bromide, 1,2-dichlorobenzene, 110 °C, 24 h; (**b**) *N,N'*-Diphenyl-formamidine, acetic anhydride, 120 °C, 0.5 h; (**c**) 4-(Bromomethyl)phenylacetic acid, 1,2-dichlorobenzene, 110 °C, 12 h; (**d**) Triethylamine, ethanol, 80 °C, 0.5 h.

(**a**) A solution of *n*-propyl bromide (0.1 mmol, 1 equiv.) and 2-methylbenzoxazole (0.1 mmol, 1 equiv.) in 1,2-dichlorobenzene (10 mL) was stirred at 110 °C for 24 h. The reaction mixture was diluted with diethyl ether, and the precipitate was washed with diethyl ether to obtain desired product **3** as a white crystalline (Yield: 19.7%).

(**b**) A solution of **3** (0.1 mmol, 1 equiv.) and *N,N'*-diphenylformamidine (0.1 mmol, 1.2 equiv.) in acetic anhydride (10 mL) was stirred at 120 °C for 30 min. The solvent was removed under reduced pressure, and the residue was purified by silica-gel flash column chromatography with dichloromethane/methanol to obtain desired product **4** as a yellow crystalline (Yield: 93.1%).

(**c**) A solution of 4-(bromomethyl)phenylacetic acid (0.1 mmol, 1 equiv.) and 2-methylbenzoxazole (0.1 mmol, 1 equiv.) in 1,2-dichlorobenzene (10 mL) was stirred at 110 °C for 12 h. The reaction mixture was diluted with diethyl ether, and the precipitate was washed with diethyl ether to obtain the desired

product **5** as a yellow crystalline (Yield: 99.9%). The characterization of **5** was previously reported.[3]

(**d**) A solution of **4** (0.1 mmol, 1 equiv.) and **5** (0.1 mmol, 1 equiv) in ethanol (10 mL) was added triethylamine, and stirred at 80 °C for 30 min. The solvent was removed under reduced pressure, and the residue extracted by chloroform, washed with brine. The organic layer was dried over anhydrous $MgSO_4$(s). The filtrate was evaporated and the resulting mixture was purified by silica-gel flash column chromatography with dichloromethane/methanol (with 0.1% acetic acid) to obtain desired product **Cy2** as a deep yellow crystalline (Yield: 54.5%).

■ 2-Methyl-3-propylbenzo[*d*]oxazol-3-ium iodide (**3**)

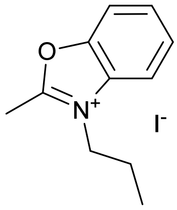

3

R_f = 0.63 (1:10 = MeOH:DCM, v/v); ^{1}H NMR (400 MHz, DMSO-d_6) δ 8.14–8.22 (m, 2H), 7.76–7.79 (m, 2H), 4.54 (t, J = 7.4 Hz, 2H), 3.11 (s, 3H), 1.90 (sextet, J = 7.2 Hz, 2H), 1.02 (t, J = 7.4 Hz, 2H); ^{13}C NMR (100 MHz, DMSO-d_6) δ 168.8, 147.4, 129.8, 128.6, 127.7, 114.7, 113.0, 47.7, 21.1, 13.5, 10.8; LRMS(ESI$^+$) m/z calcd for $C_{11}H_{14}NO$ [M-I]$^+$ 176.11; Found 176.1; HRMS(ESI$^+$) m/z calcd for $C_{11}H_{14}NO$ [M-I]$^+$ 176.1070; Found 176.1071.

■ (*E*)-2-(2-(*N*-phenylacetamido)vinyl)-3-propylbenzo[*d*]oxazol-3-ium iodide (**4**)

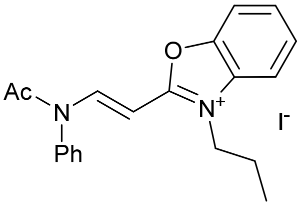

4

R_f = 0.69 (1:10 = MeOH:DCM, v/v); ^{1}H NMR (400 MHz, DMSO-d_6) δ 9.07 (d, J = 13.5 Hz, 1H), 7.99–8.09 (m, 2H), 7.64–7.73 (m, 5H), 7.51–7.54 (m, 2H), 5.46 (d, J = 13.6 Hz, 1H), 4.28 (t, J = 6.8 Hz, 2H), 2.07 (s, 3H), 1.68 (sextet, J = 7.6 Hz, 2H), 0.79 (t, J = 7.2 Hz, 3H); ^{13}C NMR (100 MHz, DMSO-d_6) δ 170.2, 163.5, 146.8, 146.7, 136.7, 130.6, 130.5, 130.2, 128.1, 128.0, 127.2, 113.7, 112.4, 87.3, 46.5, 23.3, 21.2, 10.7; LRMS(ESI$^+$) m/z calcd for $C_{20}H_{21}N_2O_2$ [M-I]$^+$ 321.16; Found 321.1.

■ 2-((1*E*,3*E*)-3-(3-(4-(carboxymethyl)benzyl)benzo[*d*]oxazol-2(3*H*)-ylidene)prop-1-en-1-yl)-3-propylbenzo[*d*] oxazol-3-ium chloride (**Cy2**)

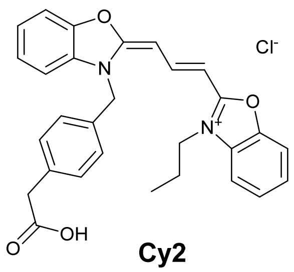

Cy2

R_f = 0.17 (1:10 = MeOH:DCM, v/v); ^{1}H NMR (400 MHz, DMSO-d$_6$) δ 8.30 (t, *J* = 13.2 Hz, 2H), 7.67–7.80 (m, 4H), 7.37–7.49 (m, 4H), 7.35 (d, *J* = 8.0 Hz, 2H), 7.28 (d, *J* = 8.4 Hz, 2H), 6.17 (d, *J* = 13.2 Hz, 1H), 6.09 (d, *J* = 13.6 Hz, 1H), 5.48 (s, 2H), 4.19 (t, *J* = 6.8 Hz, 2H), 3.54 (s, 2H), 1.79 (sextet, *J* = 7.6 Hz, 2H), 0.96 (t, *J* = 7.2 Hz, 3H); ^{13}C NMR (100 MHz, DMSO-d$_6$) δ 172.6, 172.1, 161.9, 161.9, 146.5, 146.4, 135.6, 132.3, 131.4, 131.3, 130.0, 127.2, 126.0, 126.0, 125.3, 125.1, 111.7, 111.4, 111.1, 111.0, 86.2, 84.7, 46.4, 45.2, 21.2, 21.0, 10.9; LRMS(ESI$^+$) m/z calcd for $C_{29}H_{27}N_2O_4$ [M-Cl]$^+$ 467.20; Found 467.3; HRMS(ESI$^+$) m/z calcd for $C_{29}H_{27}N_2O_4$ [M-Cl]$^+$ 467.1965; Found 467.1965.

Scheme 2. Synthesis of **Cy3**.

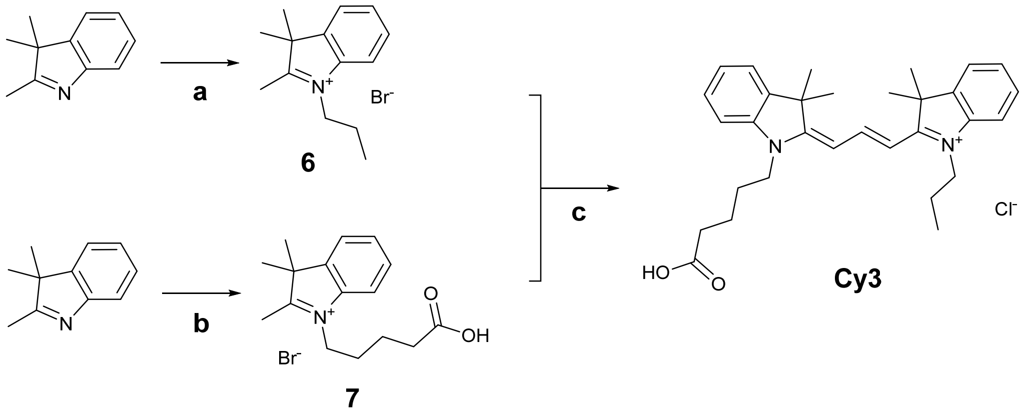

Reagents and conditions: (**a**) *n*-propyl bromide, nitromethane, 80 ˚C, 6 h; (**b**) 5-bromovaleric acid, nitromethane, 80 ˚C, 6 h; (**c**) **7**, *N,N'*-diphenylformamidine, acetic anhydride, 120 ˚C, 0.5 h; then **6**, pyridine, r. t., 12 h.

(**a**) A solution of *n*-propyl bromide (0.1 mmol, 1 equiv.) and 2,3,3-trimethylindolenine (0.1 mmol, 1 equiv.) in nitromethane (10 mL) was stirred at 80 °C for 6 h. The reaction mixture was triturated

with ether, and the precipitate was washed with ether to obtain desired product as pink crystalline (Yield: 53.1%). The characterization of **6** was previously reported.[3]

(**b**) A solution of 5-bromovaleric acid (0.1 mmol, 1 equiv.) and 2,3,3-trimethylindolenine (0.1 mmol, 1 equiv.) in nitromethane (10 mL) was stirred at 80 °C for 6 h. The reaction mixture was triturated with ether, and the precipitate was washed with ether to obtain desired product as violet crystalline (Yield: 18.5%). The characterization of **7** was previously reported.[4]

(**c**) A solution of **7** (0.1 mmol, 1 equiv.) and *N,N'*-diphenylformamidine (0.12 mmol, 1.2 equiv.) in acetic anhydride (10 mL) were stirred at 120 °C for 30 min. The reaction mixture was cooled to room temperature, then a solution of **6** in pyridine (10 mL) was added. The mixture was stirred at room temperature for 12 h. The solution was concentrated and dissolved in chloroform, and washed with water and 1N HCl aqueous solution. The organic layer was then dried with $MgSO_4$, filtered, evaporated, and purified by silica-gel flash column chromatography with dichloromethane/methanol to obtain **Cy3** as a deep pink crystalline (Yield: 34.2%).

■ 2-((*E*)-3-((*E*)-1-(4-carboxybutyl)-3,3-dimethylindolin-2-ylidene)prop-1-en-1-yl)-3,3-dimethyl-1-propyl-3*H*-indol-1-ium chloride (**Cy3**)

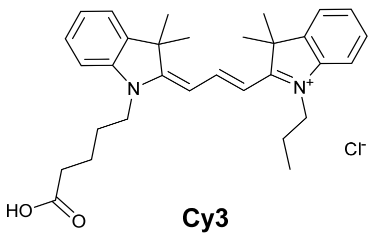

Cy3

R_f = 0.21 (1:10 = MeOH:DCM, v/v); ^{1}H NMR (400 MHz, DMSO-d_6) δ 8.37 (t, J = 13.4 Hz, 1H), 7.65 (d, J = 7.2 Hz, 2H), 7.42–7.52 (m, 4H), 7.30 (t, J = 7.4 Hz, 2H), 6.58–6.63 (m, 2H), 4.10–4.17 (m, 4H), 3.36 (brs, 1H), 2.29 (m, 2H), 1.58–1.83 (m, 18H), 1.00 (t, J = 7.2 Hz, 3H); ^{13}C NMR (100 MHz, DMSO-d_6) δ 173.9, 173.8, 149.9, 142.0, 141.9, 140.6, 128.6, 125.2, 122.9, 122.5, 118.9, 111.7, 111.6, 102.6, 79.3, 48.9, 48.9, 45.1, 43.6, 27.5, 27.5, 26.7, 22.0, 20.5, 11.0; LRMS(ESI$^+$) m/z calcd for $C_{31}H_{39}N_2O_2$ [M-Cl]$^+$ 471.30; Found 471.4; HRMS(ESI$^+$) m/z calcd for $C_{31}H_{39}N_2O_2$ [M-Cl]$^+$ 471.3006; Found 471.3005.

Scheme 3. Synthesis of **Cy5**.

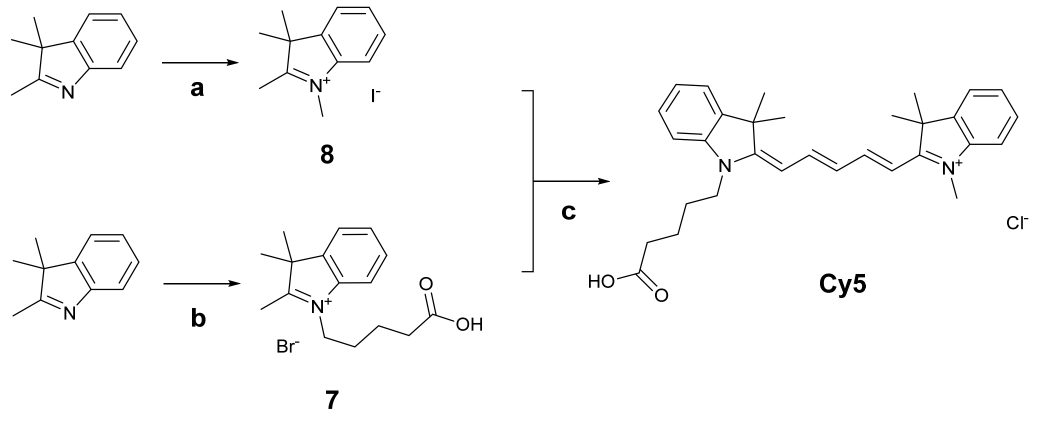

Reagents and conditions: (**a**) Iodomethane, nitromethane, r. t., 12 h; (**b**) 5-Bromovaleric acid, nitromethane, 80 °C, 6 h; (**c**) **7**, malondialdehyde bis(phenylimine) monohydrochloride, acetic anhydride, 120 °C, 0.5 h; then **8**, pyridine, r. t., 12 h.

(**a**) The preparation and characterization of **8** were previously reported.[5]

(**b**) Same as **Scheme 2b**.

(**c**) A solution of **5** (0.1 mmol, 1 equiv.) and malondialdehyde bis(phenylimine) monohydrochloride (0.12 mmol, 1.2 equiv.) in acetic anhydride (10 mL) was stirred at 120 °C for 30 min. The reaction mixture was cooled to room temperature, then a solution of **6** in pyridine (10 mL) was added. The mixture was stirred at room temperature for 12 h. The solution was concentrated and dissolved in chloroform, and washed with water and 1N HCl aqueous solution. The organic layer was then dried with $MgSO_4$(s), filtered, evaporated, and purified by flash column chromatography with dichloromethane/methanol to obtain **Cy5** as a deep blue crystalline (Yield: 50.4%). The characterization of **Cy5** was previously reported.[4]

Scheme 4. Synthesis of **Cy2**, **Cy3**, **Cy5-NHS ester**.

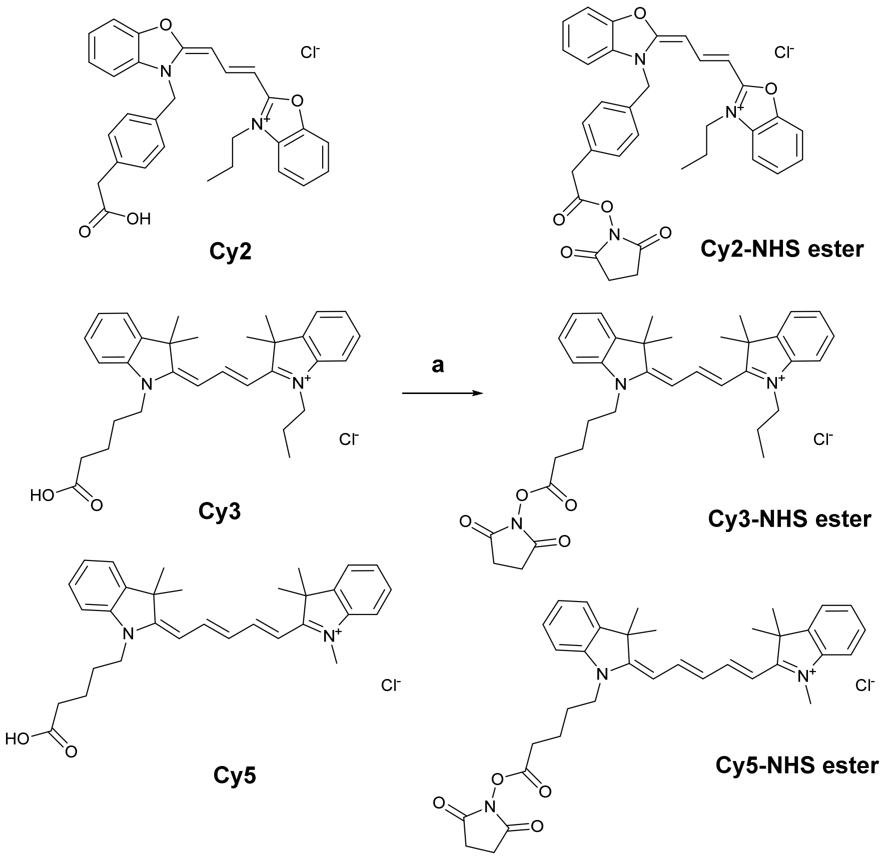

Reagents and conditions: (**a**) pyridine, DSC, DMF, 60 °C, 2 h.

(**a**) To a solution of **Cy2**, **Cy3**, or **Cy5** (0.1 mmol, 1.0 equiv.) in DMF (5 mL) was added pyridine (0.2 mL) and *N,N'*-disuccinimidyl carbonate (DSC, 0.15 mmol, 1.5 equiv.). The mixture was stirred at 60 °C for 2 h. The solvent was removed under reduced pressure, and the residue was purified by silica-gel flash column chromatography with dichloromethane/methanol to obtain desired product **Cy2-NHS ester** (Yield: 71.6%), **Cy3-NHS ester** (Yield: 79.8%), or **Cy5-NHS ester** (Yield: 80.2%), respectively.

■ 2-((1*E*,3*E*)-3-(3-(4-(2-((2,5-dioxopyrrolidin-1-yl)oxy)-2-oxoethyl)benzyl)benzo[*d*]oxazol-2(3*H*)-ylidene)prop-1-en-1-yl)-3-propylbenzo[*d*]oxazol-3-ium chloride (**Cy2-NHS ester**)

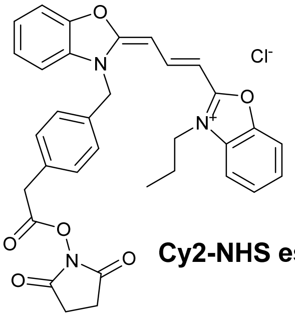

Cy2-NHS ester

R_f = 0.29 (1:10 = MeOH:DCM, v/v); ^{1}H NMR (400 MHz, $CDCl_3$) δ 8.47 (t, *J* = 13.0 Hz, 1H), 7.26–7.51 (m, 10H), 6.63–6.77 (m, 2H), 5.53–5.56 (m, 2H), 4.27 (t, *J* = 6.8 Hz, 2H), 3.67 (s, 2H), 2.03 (d, *J* = 7.2 Hz, 4H), 1.95–2.02 (m, 2H), 1.10 (t, *J* = 9.2 Hz, 2H); LRMS(ESI$^+$) m/z calcd for $C_{33}H_{30}N_3O_6$ [M-Cl]$^+$ 564.21; Found 564.2; HRMS(ESI$^+$) m/z calcd for $C_{33}H_{30}N_3O_6$ [M-Cl]$^+$ 564.2129; Found 564.2131.

■ 2-((*E*)-3-((*E*)-1-(5-((2,5-dioxopyrrolidin-1-yl)oxy)-5-oxopentyl)-3,3-dimethylindolin-2-ylidene)prop-1-en-1-yl)-3,3-dimethyl-1-propyl-3*H*-indol-1-ium chloride (**Cy3-NHS ester**)

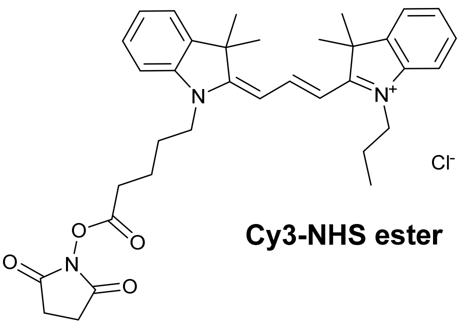

Cy3-NHS ester

R_f = 0.49 (1:10 = MeOH:DCM, v/v); ^{1}H NMR (400 MHz, DMSO-d_6) δ 8.37 (t, *J* = 18.8 Hz, 1H), 7.63–7.66 (m, 2H), 7.42–7.52 (m, 4H), 7.28–7.33 (m, 2H), 6.50–6.57 (m, 2H), 4.09–4.19 (m, 4H), 2.81 (s, 4H), 1.96 (d, *J* = 12.8 Hz, 2H), 1.69–1.84 (m, 20H), 0.99 (t, *J* = 7.4 Hz, 1H); ^{13}C NMR (100 MHz, DMSO-d_6) δ 174.1, 173.8, 170.2, 168.8, 150.0, 142.0, 141.8, 140.6, 140.6, 128.7, 125.2, 122.5, 111.7, 111.6, 102.6, 79.2, 49.0, 28.9, 29.8, 27.5, 26.1, 25.4, 21.6, 20.4, 11.0; LRMS(ESI$^+$) m/z calcd for $C_{35}H_{42}N_3O_4$ [M-Cl]$^+$ 568.32; Found 568.3; HRMS(ESI$^+$) m/z calcd for $C_{35}H_{42}N_3O_4$ [M-Cl]$^+$ 568.3170; Found 568.3168.

■ 2-((1*E*,3*E*)-5-((*E*)-1-(5-((2,5-dioxopyrrolidin-1-yl)oxy)-5-oxopentyl)-3,3-dimethylindolin-2-ylidene)penta-1,3-dien-1-yl)-1,3,3-trimethyl-3*H*-indol-1-ium chloride (**Cy5-NHS ester**)

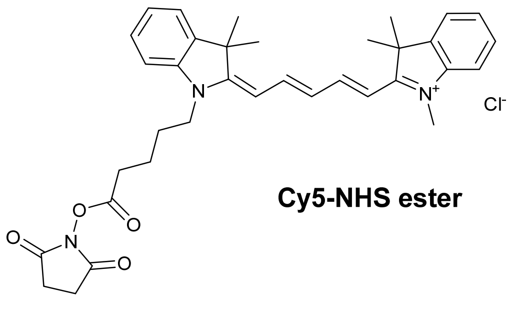

Cy5-NHS ester

R_f = 0.51 (1:10 = MeOH:DCM, v/v); ^{1}H NMR (400 MHz, DMSO-d_6) δ 8.35 (t, J = 13.2 Hz, 2H), 7.63 (d, J = 7.6 Hz, 2H), 7.37–7.42 (m, 4H), 7.22–7.29 (m, 2H), 6.58 (t, J = 12.2 Hz, 1H), 6.34 (d, J = 13.6 Hz, 1H), 6.29 (d, J = 13.6 Hz, 1H), 4.09–4.19 (m, 2H), 3.62 (s, 3H), 2.78–2.84 (m, 6H), 1.59–1.82 (m, 16H); ^{13}C NMR (100 MHz, DMSO-d_6) δ 173.4, 172.3, 170.2, 170.1, 168.8, 154.1, 154.0, 142.7, 142.0, 141.0, 141.0, 128.4, 125.4, 124.8, 124.6, 122.4, 122.3, 111.1, 111.0, 103.4, 103.0, 48.9, 48.8, 42.8, 31.2, 31.1, 29.7, 27.2, 27.0, 25.9, 25.4, 21.5; LRMS(ESI$^+$) m/z calcd for $C_{35}H_{40}N_3O_4$ $[M-Cl]^+$ 566.30; Found 566.3; HRMS(ESI$^+$) m/z calcd for $C_{35}H_{40}N_3O_4$ $[M-Cl]^+$ 566.3013; Found 566.3014.

5. Supplementary references

1 (a) N. Isakov, D. Galron, T. Mustelin, G. R. Pettit and A. Altman, *J. Immunol.*, 1993, **150**, 1195-1204; (b) B. L. Levine, W. S. May, P. G. Tyler and A. D. Hess, *J. Immunol.*, 1991, **147**, 3474-3481.

2 Martinez Molina, R. Jafari, M. Ignatushchenko, T. Seki, E. A. Larsson, C. Dan, L. Sreekumar, Y. Cao and P. Nordlund, *Science*, 2013, **341**, 84-87.

3 M. E. Jung and W.-J. Kim, *Biorg. Med. Chem.*, 2006, **14**, 92-97.

4 G. A. Korbel, G. Lalic and M. D. Shair, *J. Am. Chem. Soc.*, 2001, **123**, 361-362.

5 (a) R. L. Hinman and J. Lang, *Tetrahedron Lett.*, 1960, **1**, 12-15; (b) M. V. Kvach, A. V. Ustinov, I. A. Stepanova, A. D. Malakhov, M. V. Skorobogatyi, V. V. Shmanai and V. A. Korshun, *Eur. J. Org. Chem.*, 2008, **2008**, 2107-2117.

6. Appendices: NMR spectra

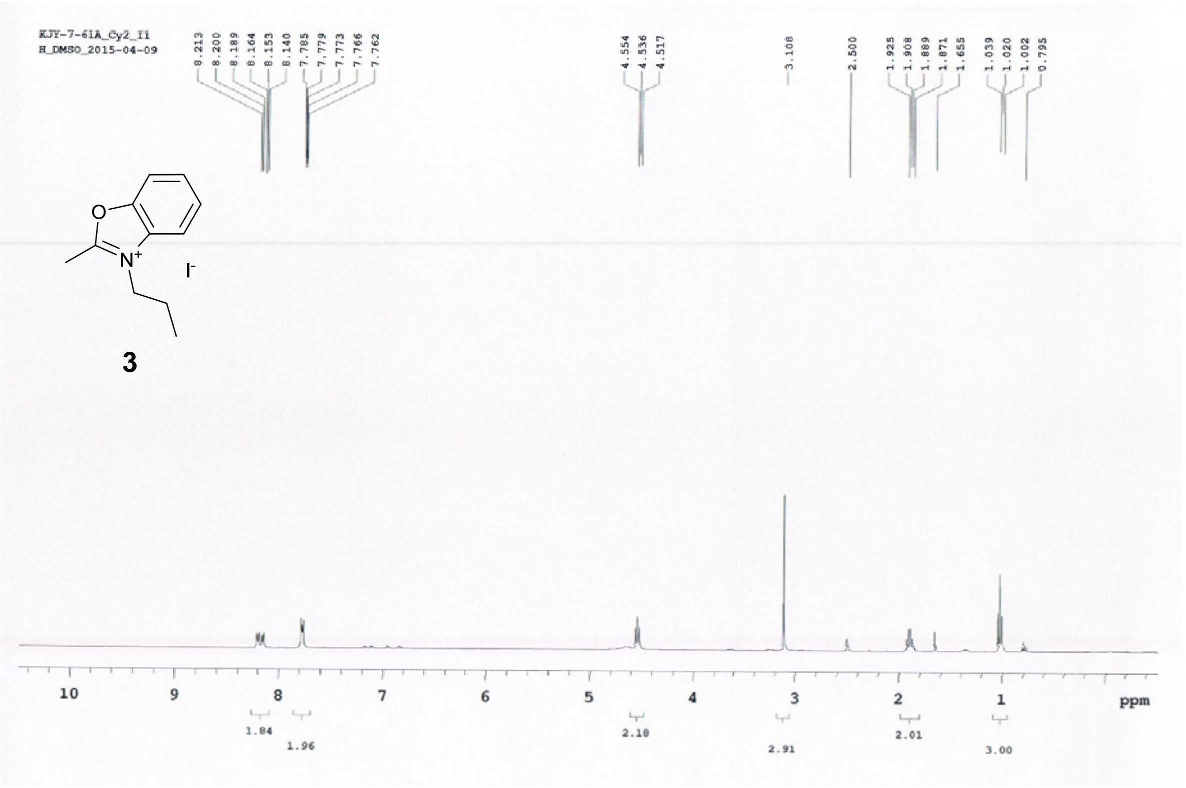

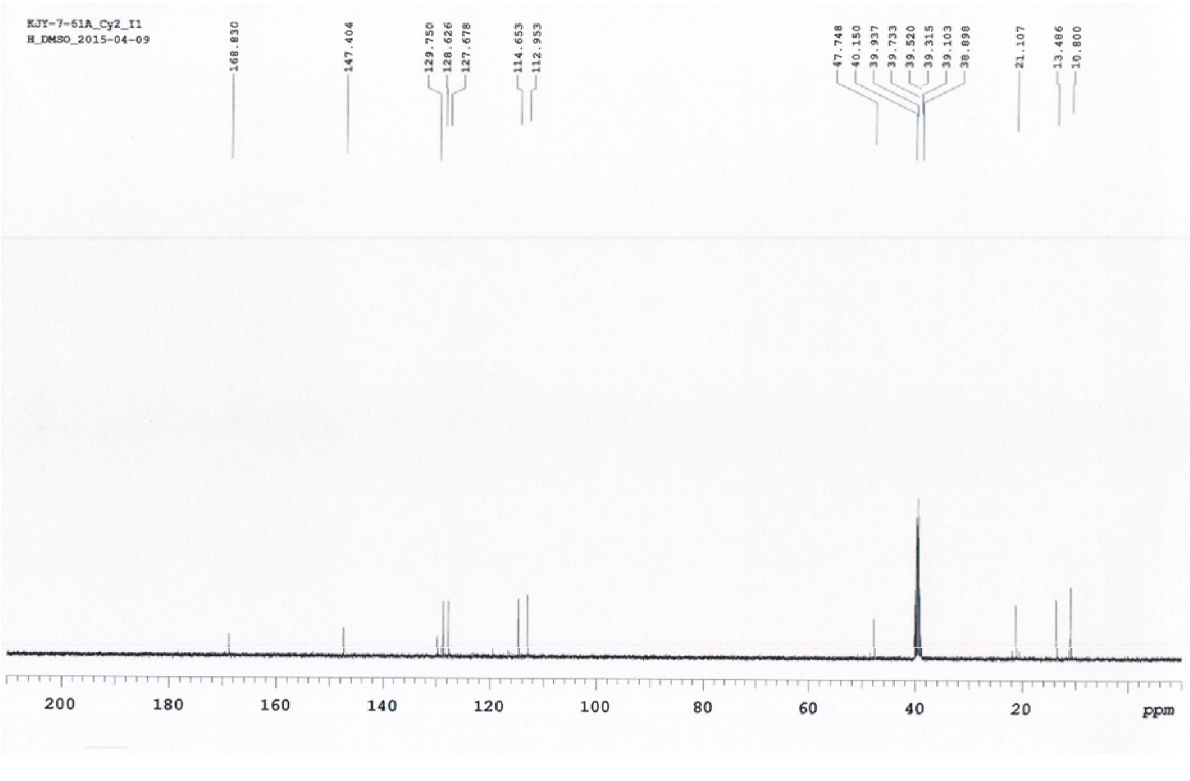

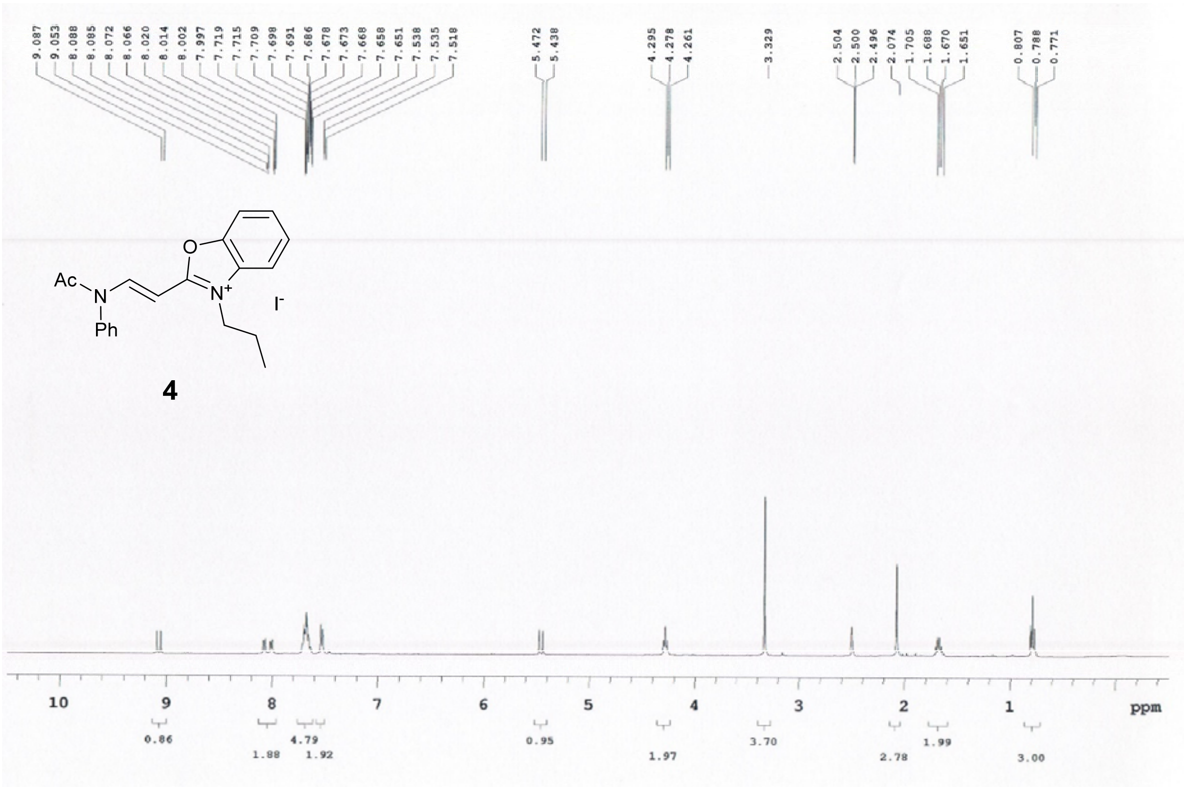
9.087 9.053 8.088 8.085 8.072 8.066 8.020 8.014 8.002 7.997 7.719 7.715 7.709 7.698 7.691 7.686 7.678 7.673 7.668 7.658 7.651 7.538 7.535 7.518
5.472 5.438
4.295 4.278 4.261
3.329
2.504 2.500 2.496 2.074 1.705 1.688 1.670 1.651
0.807 0.788 0.771
Ac
N
Ph
O
N+
I-
4
10 9 8 7 6 5 4 3 2 1 ppm
0.86 1.88 4.79 1.92 0.95 1.97 3.70 2.78 1.99 3.00

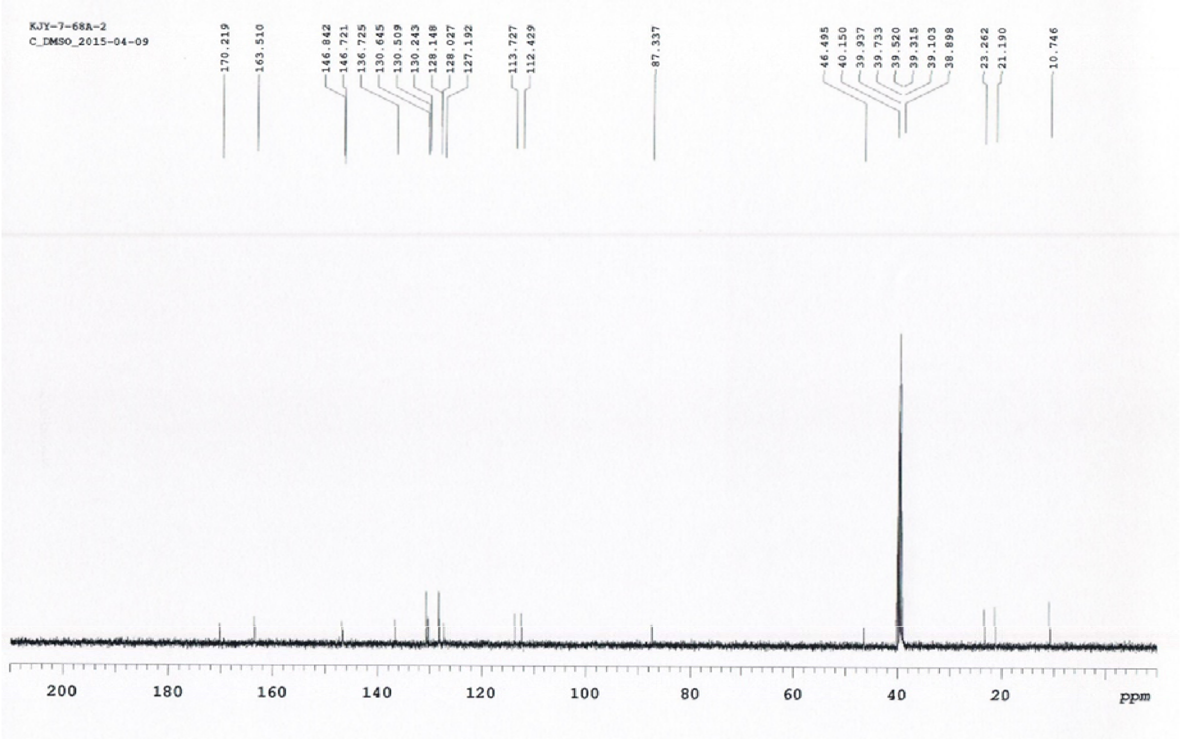
KJY-7-68A-2
C_DMSO_2015-04-09
170.219 163.510 146.842 146.721 136.725 130.645 130.509 130.243 128.148 128.027 127.192 113.727 112.429
87.337
46.495 40.150 39.937 39.733 39.520 39.315 39.103 38.898 23.262 21.190 10.746
200 180 160 140 120 100 80 60 40 20 ppm

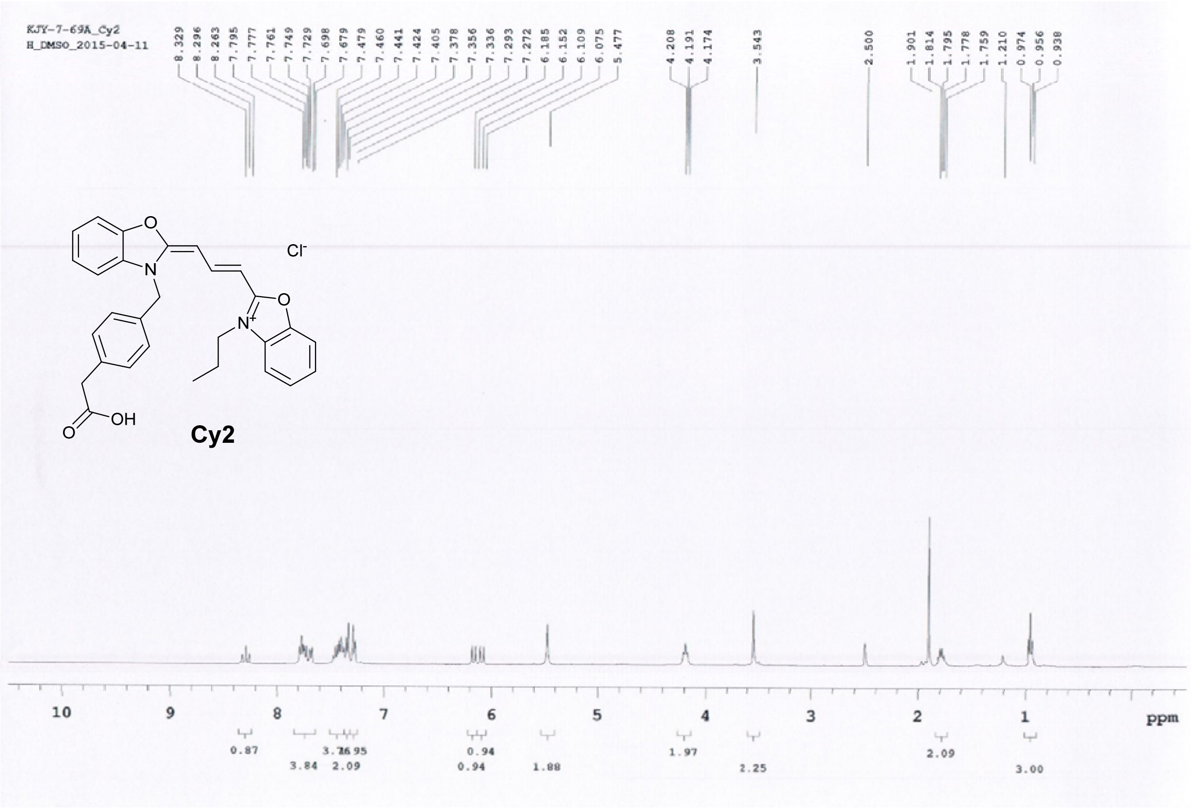
KJY-7-69A_Cy2
H_DMSO_2015-04-11
8.329 8.296 8.263 7.795 7.777 7.761 7.749 7.729 7.698 7.679 7.479 7.460 7.441 7.424 7.405 7.378 7.356 7.336 7.293 7.272 6.185 6.152 6.109 6.075 5.477
4.208 4.191 4.174
3.543
2.500
1.901 1.814 1.795 1.778 1.759 1.210 0.974 0.956 0.938
O
N
Cl-
O
N+
OH
O
Cy2
10 9 8 7 6 5 4 3 2 1 ppm
0.87
3.84 2.09
0.94
0.94 1.88
1.97
2.25
2.09
3.00

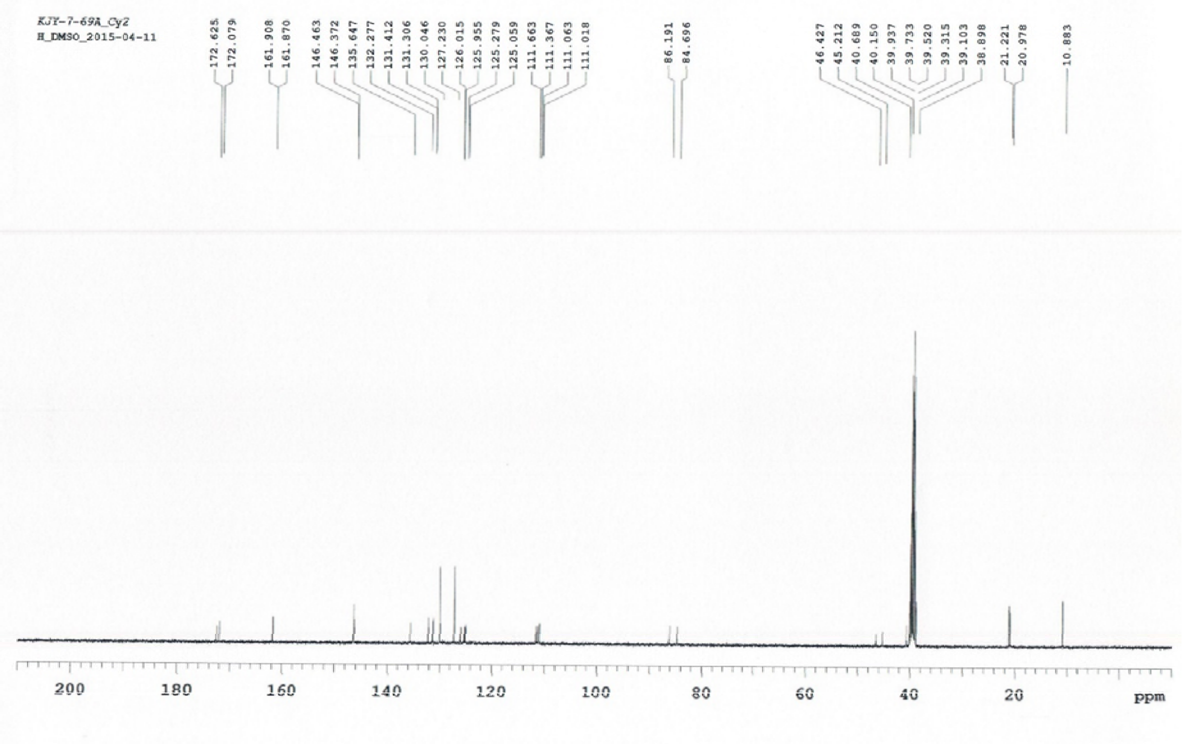
KJY-7-69A_Cy2
H_DMSO_2015-04-11
172.625 172.079 161.908 161.870 146.463 146.372 135.647 132.277 131.412 131.306 130.046 127.230 126.015 125.955 125.279 125.059 111.663 111.367 111.063 111.018
86.191 84.696
46.427 45.212 40.689 40.150 39.937 39.733 39.520 39.315 39.103 38.898 21.221 20.978 10.883
200 180 160 140 120 100 80 60 40 20 ppm

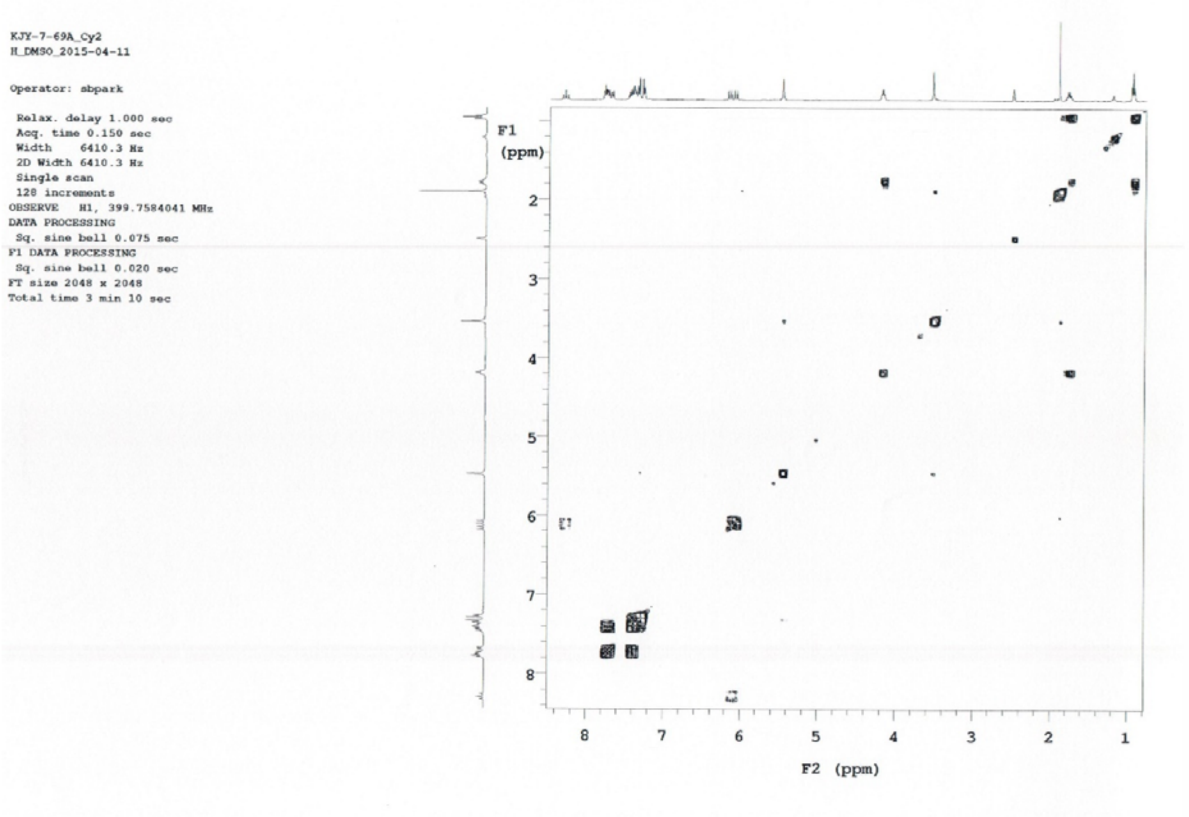
KJY-7-69A_Cy2
H_DMSO_2015-04-11
Operator: sbpark
Relax. delay 1.000 sec
Acq. time 0.150 sec
Width 6410.3 Hz
2D Width 6410.3 Hz
Single scan
128 increments
OBSERVE H1, 399.7584041 MHz
DATA PROCESSING
Sq. sine bell 0.075 sec
F1 DATA PROCESSING
Sq. sine bell 0.020 sec
FT size 2048 x 2048
Total time 3 min 10 sec
F1 (ppm)
F2 (ppm)

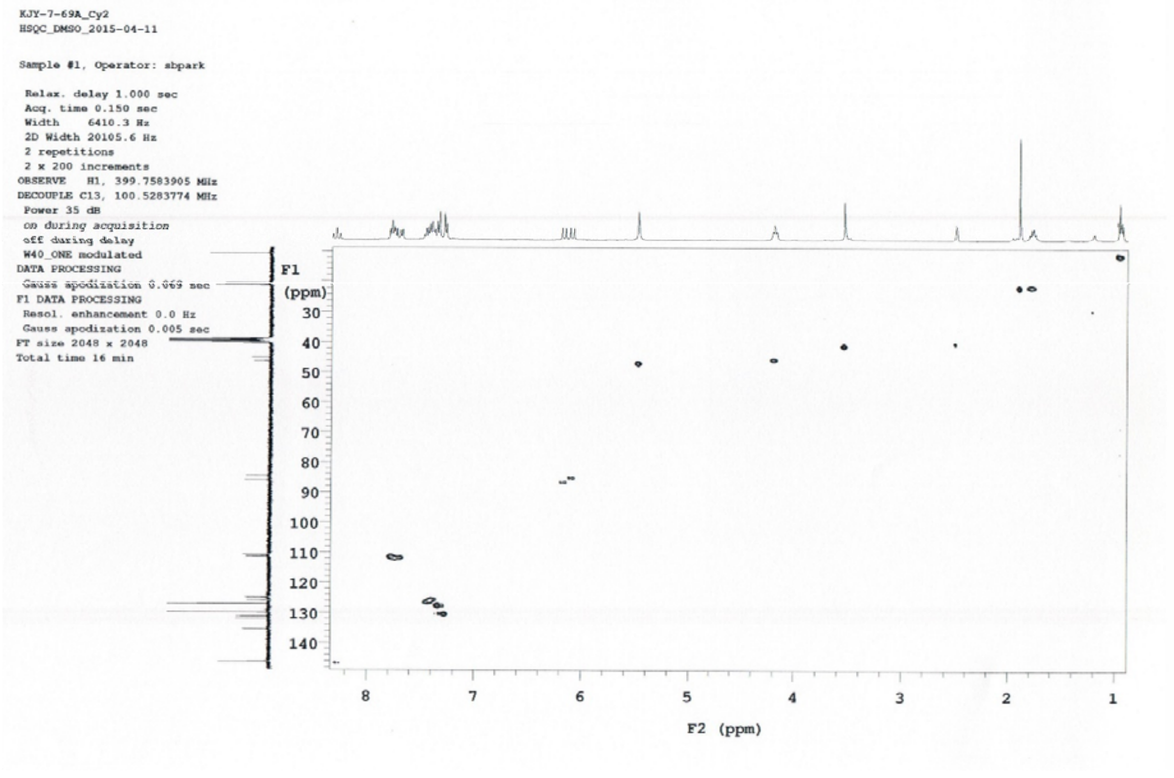
KJY-7-69A_Cy2
HSQC_DMSO_2015-04-11
Sample #1, Operator: sbpark
Relax. delay 1.000 sec
Acq. time 0.150 sec
Width 6410.3 Hz
2D Width 20105.6 Hz
2 repetitions
2 x 200 increments
OBSERVE H1, 399.7583905 MHz
DECOUPLE C13, 100.5283774 MHz
Power 35 dB
on during acquisition
off during delay
W40_ONE modulated
DATA PROCESSING
Gauss apodization 0.069 sec
F1 DATA PROCESSING
Resol. enhancement 0.0 Hz
Gauss apodization 0.005 sec
FT size 2048 x 2048
Total time 16 min
F1 (ppm)
F2 (ppm)

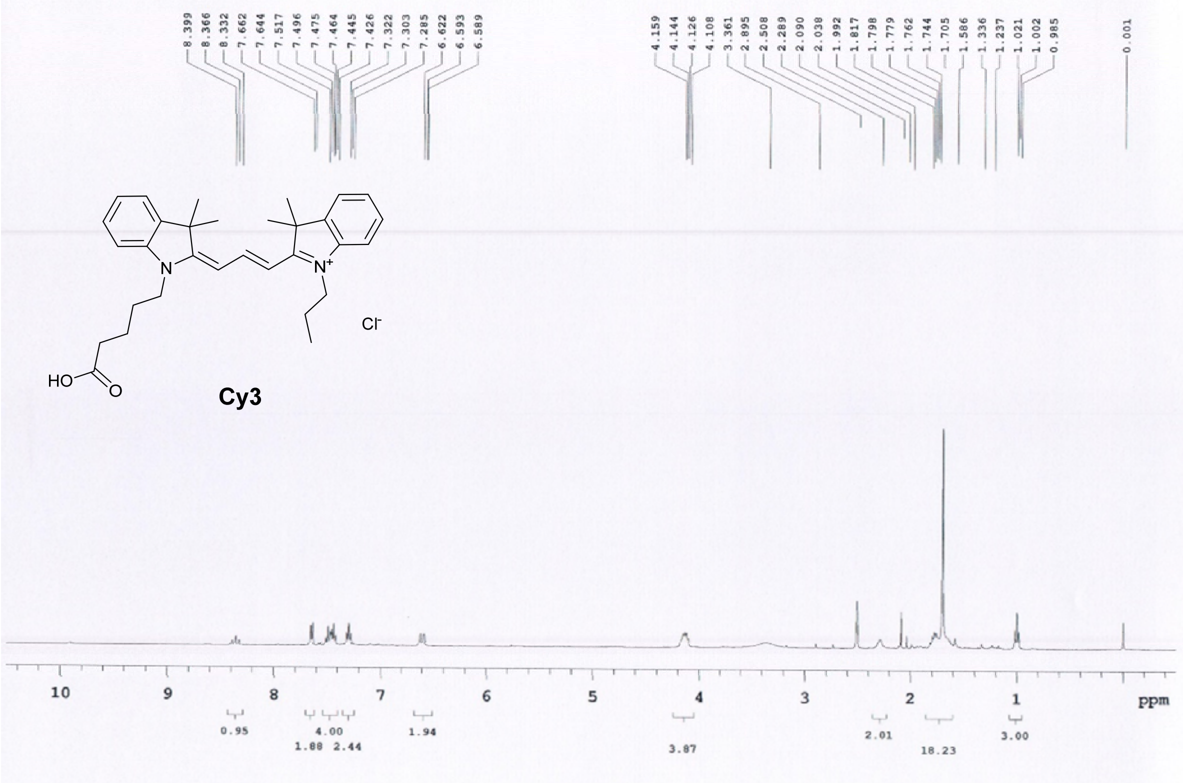
8.399
8.366
8.332
7.662
7.644
7.517
7.496
7.475
7.464
7.445
7.426
7.322
7.303
7.285
6.622
6.593
6.589
4.159
4.144
4.126
4.108
3.361
2.895
2.508
2.289
2.090
2.038
1.992
1.817
1.798
1.779
1.762
1.744
1.705
1.586
1.336
1.237
1.021
1.002
0.985
0.001
N
N+
Cl-
HO
O
Cy3
10
9
8
7
6
5
4
3
2
1
ppm
0.95
4.00
1.88
2.44
1.94
3.87
2.01
18.23
3.00

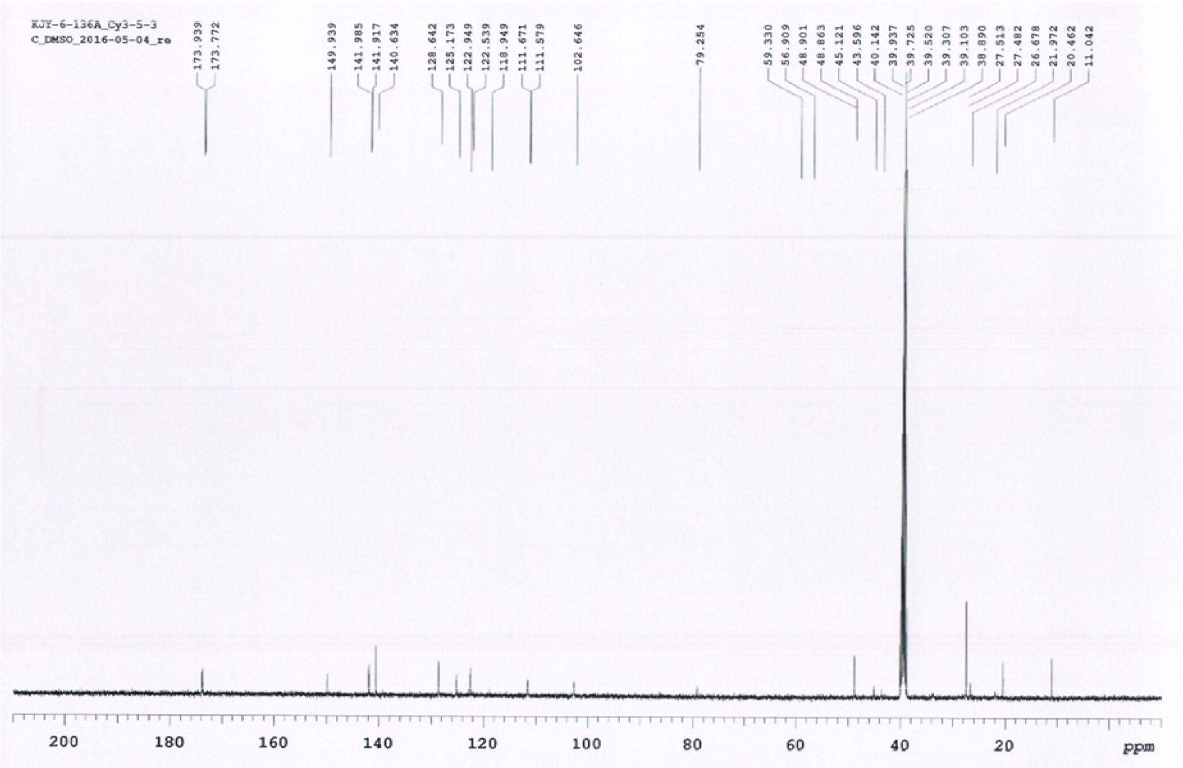
KJY-6-136A_Cy3-5-3
C_DMSO_2016-05-04_re
173.939
173.772
149.939
141.985
141.917
140.634
128.642
125.173
122.949
122.539
118.949
111.671
111.579
102.646
79.254
59.330
56.909
48.901
48.863
45.121
43.596
40.142
39.937
39.725
39.520
39.307
39.103
38.890
27.513
27.482
26.678
21.972
20.462
11.042
200
180
160
140
120
100
80
60
40
20
ppm

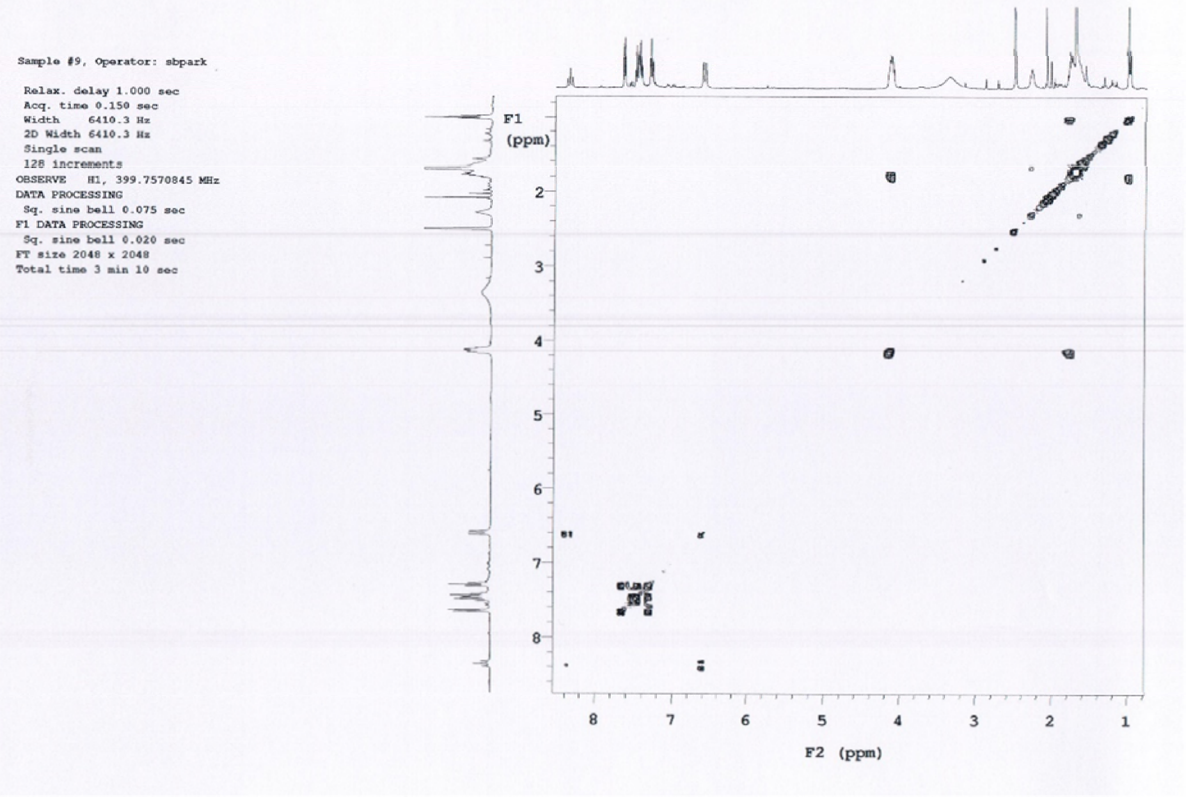
Sample #9, Operator: sbpark
Relax. delay 1.000 sec
Acq. time 0.150 sec
Width 6410.3 Hz
2D Width 6410.3 Hz
Single scan
128 increments
OBSERVE H1, 399.7570845 MHz
DATA PROCESSING
Sq. sine bell 0.075 sec
F1 DATA PROCESSING
Sq. sine bell 0.020 sec
FT size 2048 x 2048
Total time 3 min 10 sec
F1 (ppm)
F2 (ppm)

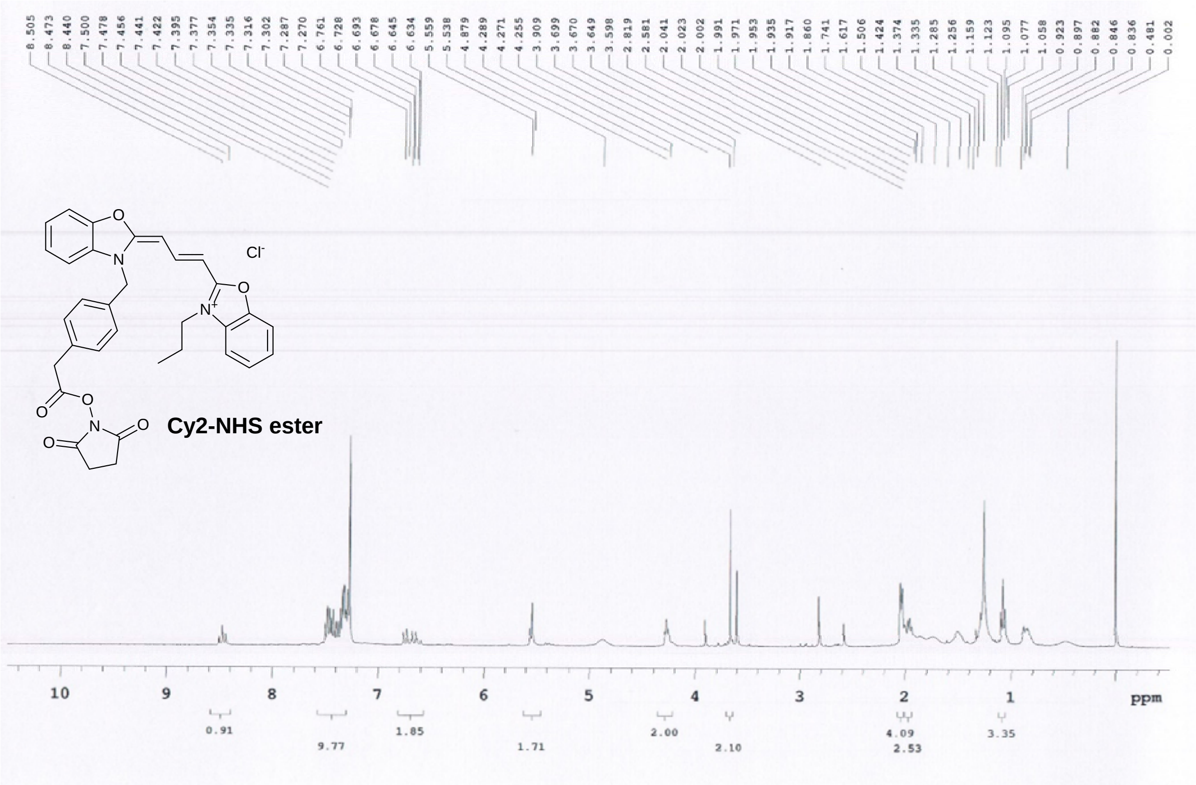
Cl-
Cy2-NHS ester
ppm
0.91
9.77
1.85
1.71
2.00
2.10
4.09
2.53
3.35

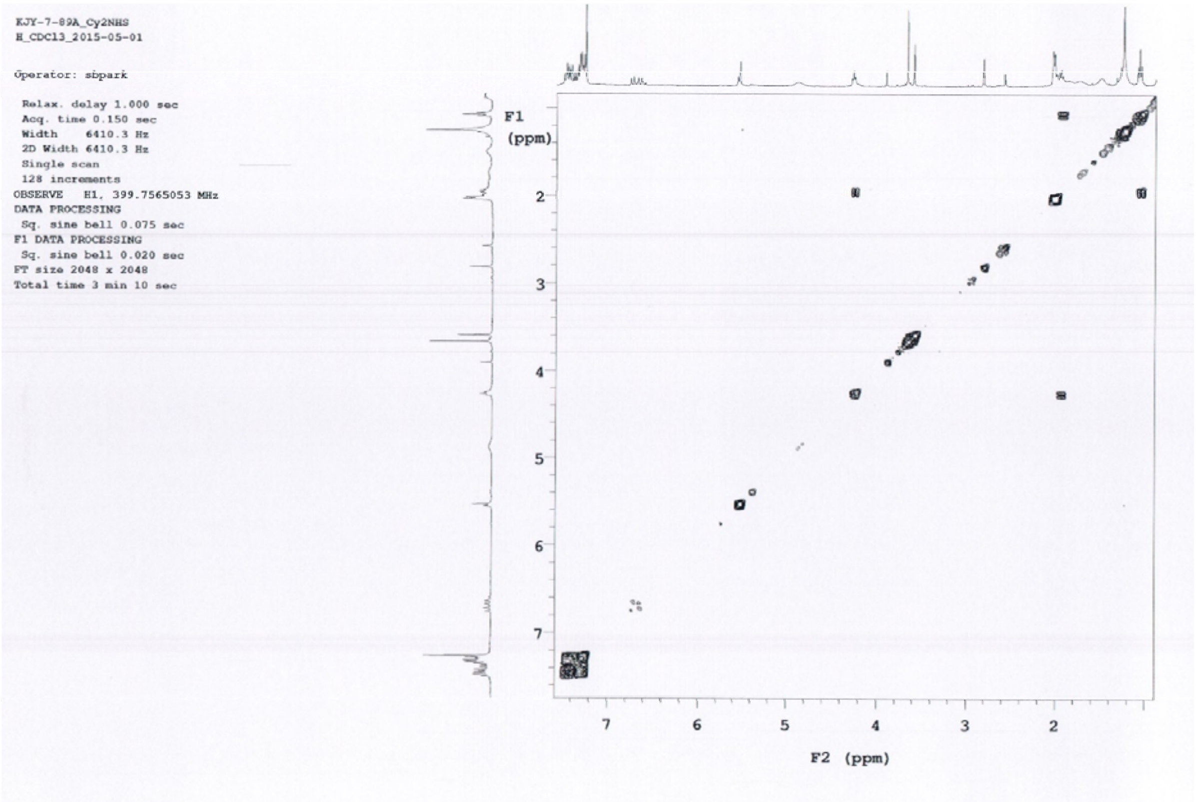
KJY-7-89A_Cy2NHS
H_CDCl3_2015-05-01
Operator: sbpark
Relax. delay 1.000 sec
Acq. time 0.150 sec
Width 6410.3 Hz
2D Width 6410.3 Hz
Single scan
128 increments
OBSERVE H1, 399.7565053 MHz
DATA PROCESSING
Sq. sine bell 0.075 sec
F1 DATA PROCESSING
Sq. sine bell 0.020 sec
FT size 2048 x 2048
Total time 3 min 10 sec
F1 (ppm)
F2 (ppm)

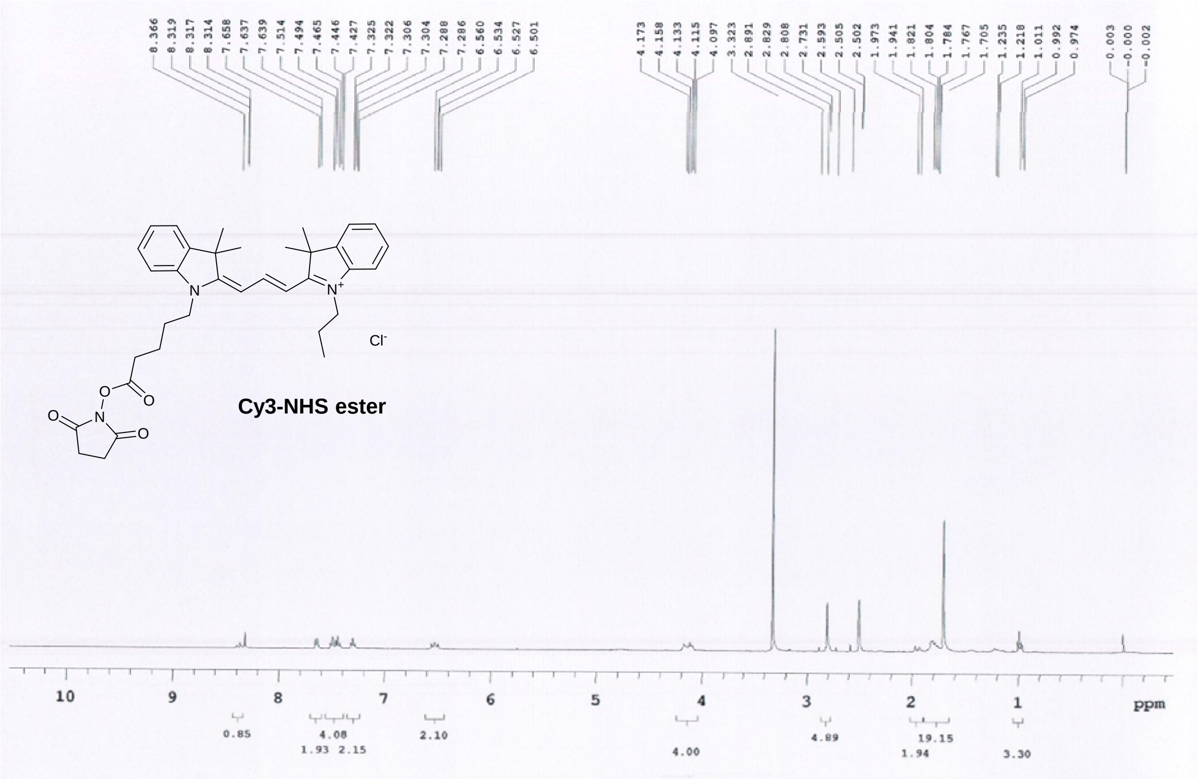
Cl⁻
Cy3-NHS ester
ppm

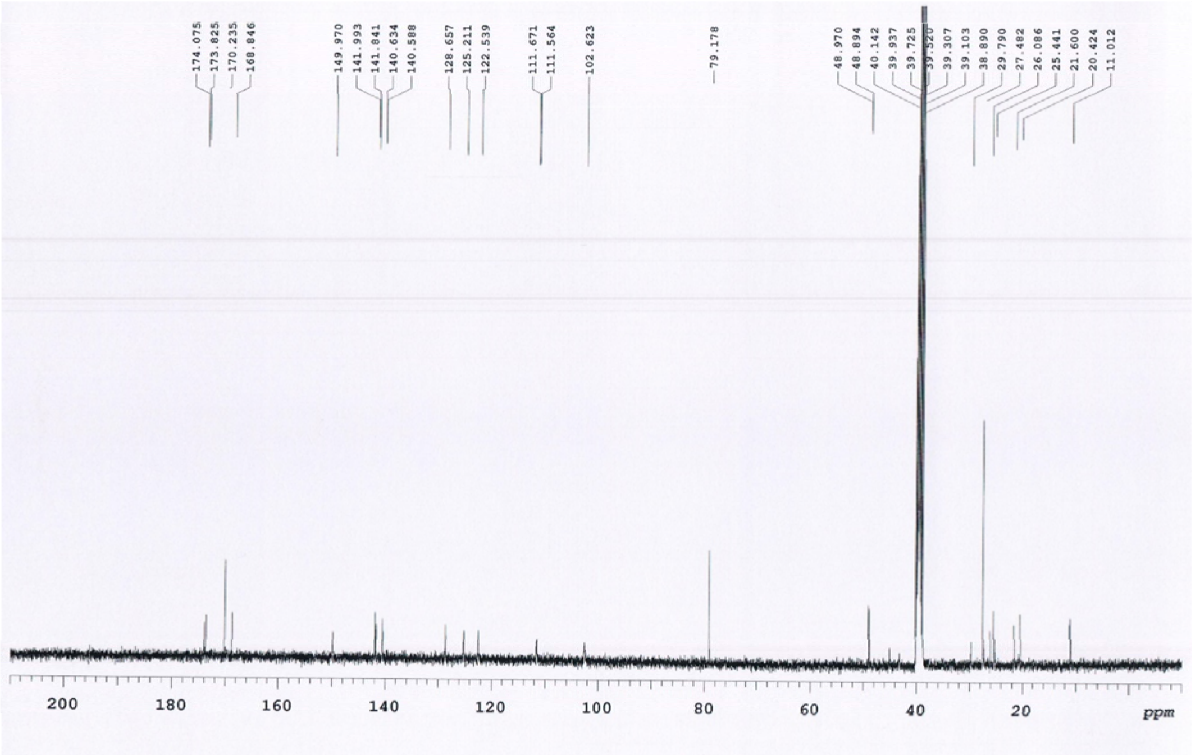
ppm

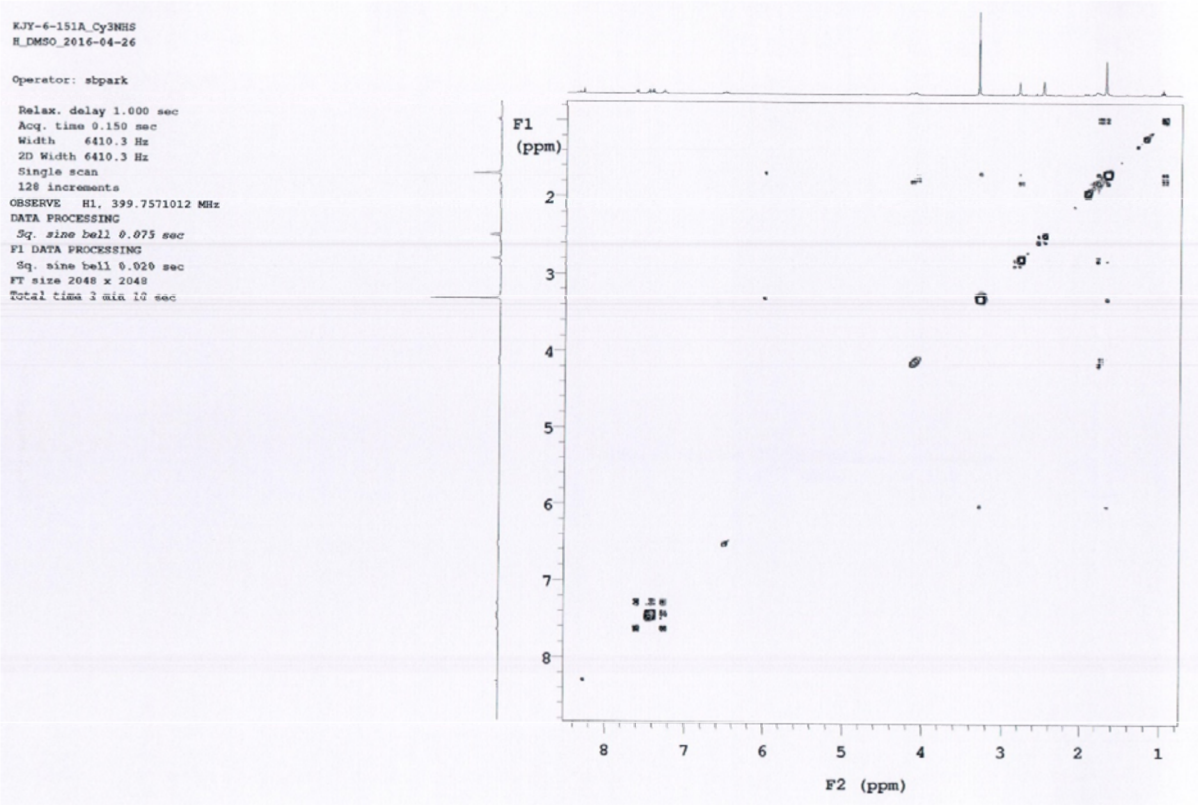
KJY-6-151A_Cy3NHS
H_DMSO_2016-04-26
Operator: sbpark
Relax. delay 1.000 sec
Acq. time 0.150 sec
Width 6410.3 Hz
2D Width 6410.3 Hz
Single scan
128 increments
OBSERVE H1, 399.7571012 MHz
DATA PROCESSING
Sq. sine bell 0.075 sec
F1 DATA PROCESSING
Sq. sine bell 0.020 sec
FT size 2048 x 2048
Total time 3 min 10 sec
F1 (ppm)
F2 (ppm)

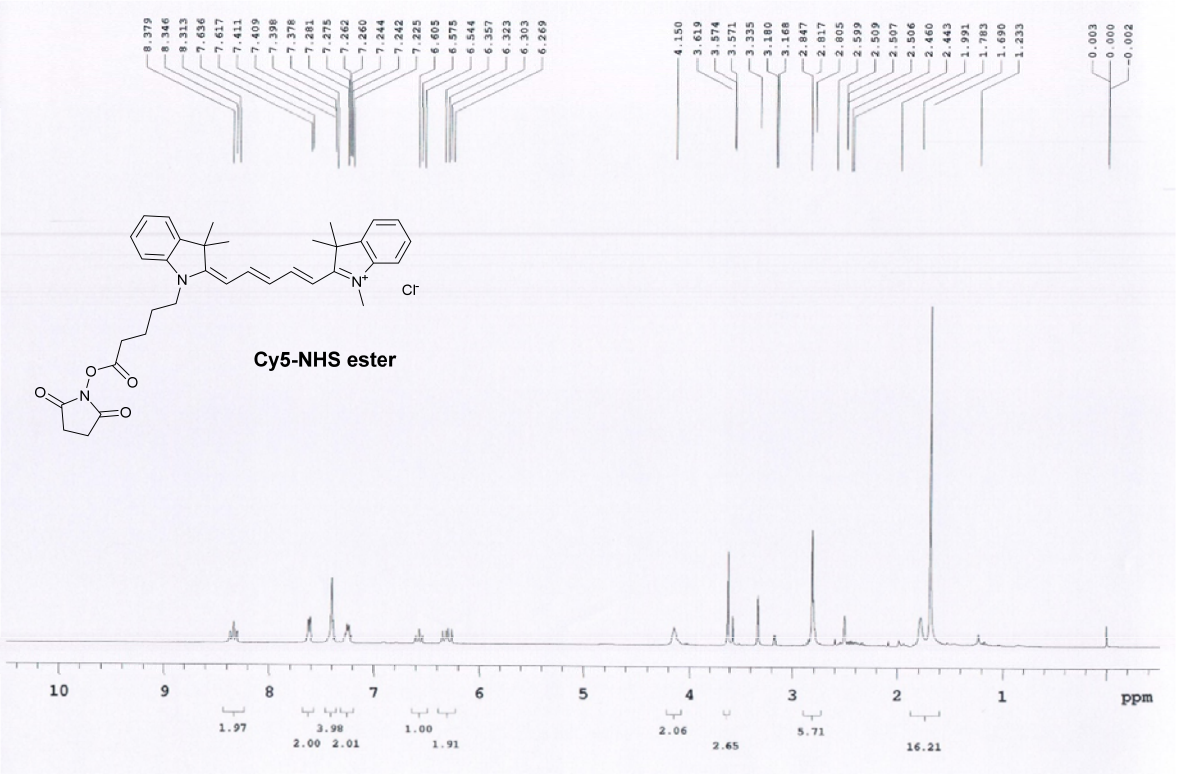
8.379
8.346
8.313
7.636
7.617
7.411
7.409
7.398
7.378
7.281
7.275
7.262
7.260
7.244
7.242
7.225
6.605
6.575
6.544
6.357
6.323
6.303
6.269
4.150
3.619
3.574
3.571
3.335
3.180
3.168
2.847
2.817
2.805
2.599
2.509
2.507
2.506
2.460
2.443
1.991
1.783
1.690
1.233
0.003
0.000
-0.002
Cl⁻
Cy5-NHS ester
10
9
8
7
6
5
4
3
2
1
ppm
1.97
3.98
2.00
2.01
1.00
1.91
2.06
2.65
5.71
16.21

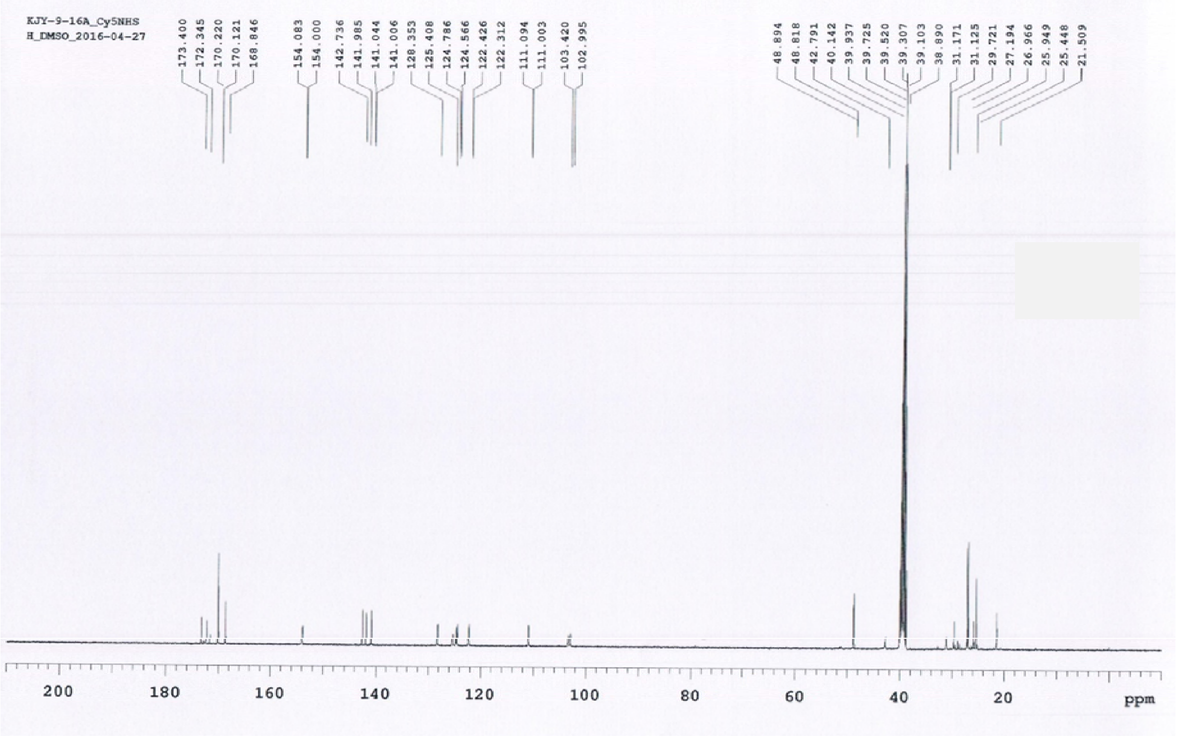
KJY-9-16A_Cy5NHS
H_DMSO_2016-04-27
173.400
172.345
170.220
170.121
168.846
154.083
154.000
142.736
141.985
141.044
141.006
128.353
125.408
124.786
124.566
122.426
122.312
111.094
111.003
103.420
102.995
48.894
48.818
42.791
40.142
39.937
39.725
39.520
39.307
39.103
38.890
31.171
31.125
29.721
27.194
26.966
25.949
25.448
21.509
200
180
160
140
120
100
80
60
40
20
ppm

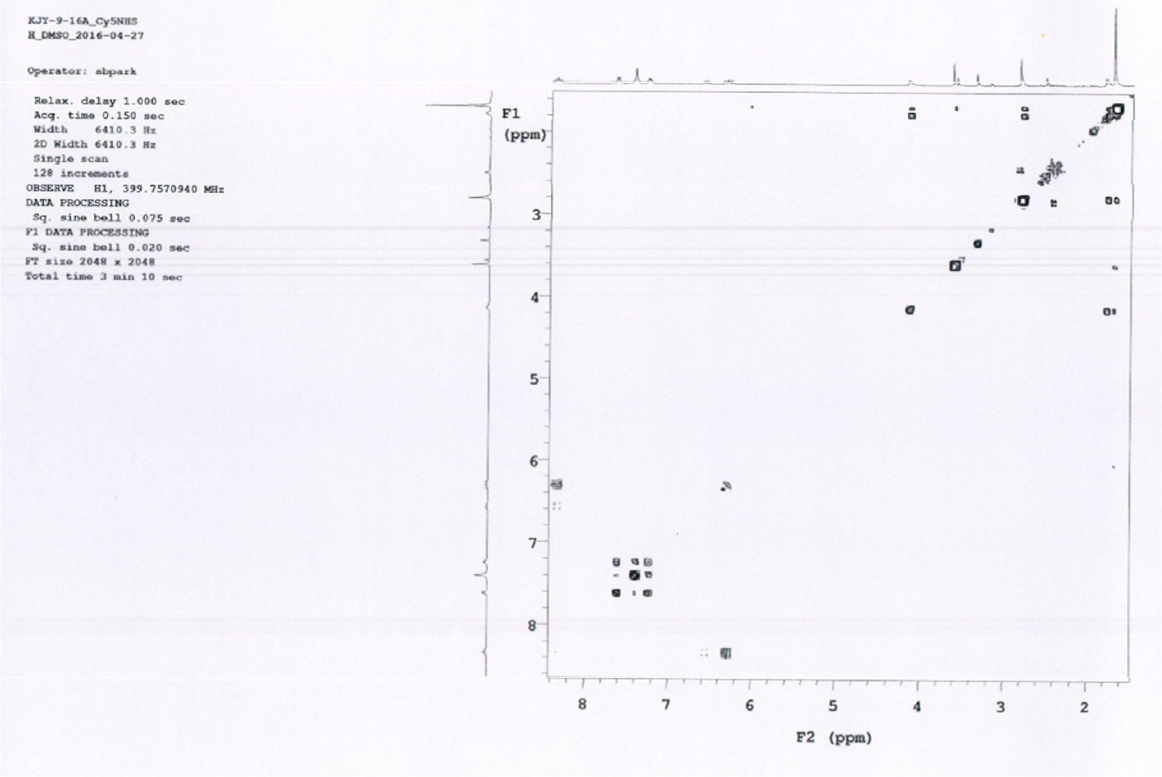
KJY-9-16A_Cy5NHS
H_DMSO_2016-04-27
Operator: sbpark
Relax. delay 1.000 sec
Acq. time 0.150 sec
Width 6410.3 Hz
2D Width 6410.3 Hz
Single scan
128 increments
OBSERVE H1, 399.7570940 MHz
DATA PROCESSING
Sq. sine bell 0.075 sec
F1 DATA PROCESSING
Sq. sine bell 0.020 sec
FT size 2048 x 2048
Total time 3 min 10 sec
F1 (ppm)
F2 (ppm)
